# From Peptide to Protein: Development of Conversion Factors for the Quantification of Gluten Using Targeted Mass Spectrometry

**DOI:** 10.1021/acs.jafc.4c12344

**Published:** 2025-05-22

**Authors:** Qianying Xu, Matthew Daly, Chiara Nitride, Olivier Tranquet, Adrian Rogers, Joan Bartra-Tomas, Angela Simpson, Peter R. Shewry, Lee A. Gethings, E. N. Clare Mills

**Affiliations:** † Division of Immunology, Immunity to Infection and Respiratory Medicine, School of Biological Sciences, Manchester Institute of Biotechnology, 5292 University of Manchester, Manchester M1 7DN, United Kingdom; ‡ Waters Corporation, Wilmslow SK9 4AX, United Kingdom; § Department of Agricultural Sciences, University of Naples Federico II, 80055 Portici, Italy; ∥ INRAE, UMR1163 Biodiversité Et Biotechnologie Fongiques, (BBF), UMR1163, Aix Marseille University, 13009 Marseille, France; ⊥ Bio-Check, St. Asaph, Denbighshire LL17 0JA, United Kingdom; # Allergy Department, Hospital Clinic, 16724 University of Barcelona, 08036 Barcelona, Spain; ∇ Clinical and Experimental Respiratory Immunoallergy, Institut Investigacions Biomediques August Pi I Sunyer (IDIBAPS), 08036 Barcelona, Spain; ○ Division of Immunology, Immunity to Infection and Respiratory Medicine, School of Biological Sciences, Manchester University NHS Foundation Trust, University of Manchester, Manchester M23 9LT, United Kingdom; ◆ 15552 Rothamsted Research, Harpenden AL5 2JQ, United Kingdom; ¶ School of Biosciences, 3660 University of Surrey, Guildford GU2 7XH, United Kingdom

**Keywords:** wheat, gluten, celiac disease, IgE reactivity, targeted mass spectrometry, conversion factor

## Abstract

Immuno- and mass spectrometry (MS) test methods have been used to ensure “gluten-free” food products contain less than 20 ppm of gluten. However, comparison of test method performance is difficult due to differences in reporting units. A set of wheat flour fractions was prepared and characterized regarding immunoglobulin E (IgE)-reactivity and protein profile, which were then used to screen a panel of gluten peptides to identify reporters suitable for use in an MS test method for gluten determination. Four peptide markers were selected and synthesized as heavy isotopically labeled versions for further evaluation. Two were derived from α-gliadin (RPQQPYPQPQPQY and QPFPQPQLPY [spanning a celiac toxic motif]), one each from γ-gliadin (GIIQPQQPAQL [spanning a celiac toxic motif and IgE epitope]), and a low-molecular-weight subunit of glutenin (VQQQIPVVQPSIL). Analysis of the wheat flour fractions was achieved with peptides RPQQPYPQPQPQY, GIIQPQQPAQL, and VQQQIPVVQPSIL. Two methods were used to derive a set of factors for converting from peptide marker to gluten protein: one based on calculation and a second on experimental analysis using either the gliadin or glutenin protein fractions. Experimentally derived conversion factors performed better when used in an MS test method to quantify gluten in a set of wheat flour samples. Peptide VQQIPVVQPSIL showed the greatest sensitivity and, when employing a glutenin fraction-based conversion factor, gave comparable results to protein levels determined using Dumas total nitrogen analysis. This peptide marker demonstrated the potential to determine gluten at a level around the 10 mg gluten/kg food product level, showing that the prototype method and approaches described have the potential to deliver a complementary method for determination of gluten in food.

## Graphical abstract

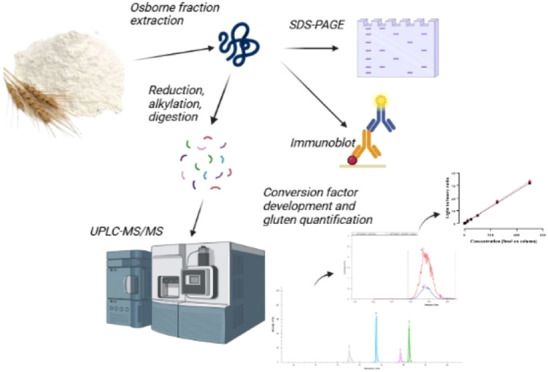

## 1 Introduction

A small proportion of the population experiences immune-mediated adverse reactions to foods derived from wheat and related cereals belonging to the *Triticeae* tribe. These reactions include the gluten intolerance syndrome, celiac disease (CD), which affects more than 1% of the global population,^1^ and immunoglobulin E (IgE)-mediated wheat allergy, which affects about 0.2% of the adult population.^2^ Celiac disease is triggered by exposure to the seed storage prolamins of wheat, barley, rye, and, less commonly, oats, which all have toxic motifs that comprise nine or more amino acid residues and are resistant to digestive proteases.^3^ Specific glutamine residues within these motifs are deamidated in the gut mucosal wall by tissue transglutaminase, the deamidated peptides being able to trigger a specific T-cell immune reaction as a consequence of binding to HLA-DQ2/8 molecules on antigen-presenting cells.^4^ The major cereal allergens triggering IgE-mediated food allergies include gluten proteins, such as ω5-gliadin (Tri a 19), α/β-gliadin (Tri a 21), γ-gliadin (Tri a 20), and a LMW glutenin subunit (Tri a 36), which are often associated with wheat-dependent exercise-induced anaphylaxis (WDEIA).^5^ In contrast, IgE-mediated wheat allergies caused by inhalation of flour particles, such as bakers’ asthma, are triggered mainly by proteins soluble in water or dilute salt solutions, such as the α-amylase/trypsin inhibitors CM3, CM16, and 0.28.^6^

Currently, there is no cure for either CD or IgE-mediated gluten allergies, and consequently, individuals with these conditions must avoid cereals containing gluten in their daily diet. The Codex Alimentarius Commission recommends that a “gluten-free” label may be used on food products that have less than 20 mg gluten/kg.^7^ Managing such “free-from” products requires effective allergen management, including analysis of gluten in raw materials and finished food products, as well as validating cleaning protocols. The simplest and most widely used method for gluten determination is an enzyme-linked immunosorbent assay (ELISA), which primarily targets the gliadin fraction extracted in aqueous ethanol mixtures. However, there are concerns that such methods can both over- and underestimate the gluten content of foods and may give inconsistent results.^8^ It is also unclear how different test methods may perform when applied to different gluten fractions; the majority of efforts focus on the gliadin fraction, which is soluble in aqueous ethanol, as indicated by the Codex recommendations.^7^ Mass spectrometry (MS) has the potential to provide a complementary and confirmatory analysis.^9,10^ However, there is a need to convert from peptide measurement to gluten protein to deliver a meaningful test result in milligrams of gluten per kg of food. One way to address this is to use a reference material, which has been shown to allow the harmonization of gluten measurements made using different ELISA test kits.^8^ Currently, the most widely used reference material for gluten detection is the Prolamin Working Group (PWG)-gliadin,^11^ which was extracted from a flour milled from a mixture of 28 various wheat cultivars and has been used as a calibrant for a great number of gluten detection methodologies. However, the PWG-gliadin is a finite resource, and it is difficult to maintain a stable supply.^12^ Furthermore, it comprised wheat varieties that are no longer commercially relevant, and it was extracted from flour using 60–70% (v/v) ethanol and therefore comprises mainly monomeric gliadin proteins. An alternative reference material from MoniQA is being developed, which may address these issues,^13^ but is not currently available.

Therefore, to develop conversion factors for a targeted MS method for the determination of gluten in food, a set of protein fractions of wheat flour was prepared using the classical Osborne fractionation procedure.^14^ These were characterized using a combination of chromatographic and immunoblotting methods employing animal antibody preparations, including those used in gluten ELISA test kits. To ensure the fractions contained relevant IgE-reactive allergen, their IgE-binding capacity was verified using wheat allergic patients’ sera. The fractions were then used to generate conversion factors for a suite of candidate peptide markers for use in a targeted mass-spectrometry-based method for quantification of gluten. Four peptide markers were selected, synthesized with a heavy isotope label, and applied to the analysis of wheat flour extracts.

## 2 Materials and Methods

### 2.1 Materials

All reagents used were of analytical grade unless otherwise specified. Bread wheat (Triticum aestivum cv. Hereward) was provided by Rothamsted Research (Hertfordshire, U.K.) and milled at Campden BRI (Gloucestershire, U.K.) with a Buhler MLU-202 Laboratory Flour Mill (Urzwill, Switzerland). Gluten-free flour was obtained from a retail outlet. Whatman grade 1 Filter paper, skim milk powder, and baker’s yeast (Saccharomyces cerevisiae) enolase were purchased from Sigma-Aldrich (Dorset, U.K.). SnakeSkin Dialysis tubing with 3.5k molecular weight cutoff (MWCO), bovine serum albumin (BSA) standard (2 mg/mL), 1-step nitroblue tetrazolium (NBT)/5-bromo-4-chloro-3-indolyl-1-phosphate (BCIP) substrate solution, PageRuler prestained protein ladder, and secondary enzyme-labeled antibodies were purchased from ThermoFisher Scientific (Hertfordshire, U.K.). Mark12 unstained standard, SeeBlue prestained protein standard, NuPAGE lithium dodecyl-sulfate (LDS) sample buffer (4×), NuPAGE 4–12% Bis–Tris gels, NuPAGE MES SDS running buffer (20×), and Simplyblue stain were all from Invitrogen, ThermoFisher Scientific (Hertfordshire, U.K.). RC DC Protein Assay, Extra thick blot filter paper, and 0.2 μm pore size nitrocellulose membrane were from Bio-Rad (Hertfordshire, U.K.). The high-performance liquid chromatography (HPLC), liquid chromatography mass spectrometry (LC–MS) grade acetonitrile, formic acid (FA), and water, along with the 0.45 μm Sartorius Ministart syringe filters, were all purchased from Fisher Scientific (Hertfordshire, U.K.). RapiGest SF and SepPak C18 cartridges were purchased from Waters (Wilmslow, U.K.). Primary and secondary animal antibody preparations were sourced and summarized in Table S1. Unlabeled light peptides were synthesized by JPT Peptide Technology (Berlin, Germany), and heavy peptides, with either Tyr or Leu residues, were ^13^C- and ^15^N-labeled, which were purchased from Biosynth Ltd. (Berkshire, U.K.).

### 2.2 Human Allergic Sera

Serum samples from 23 individuals with documented IgE-mediated food allergies to wheat were obtained from the Manchester Allergy, Respiratory and Thoracic Surgery (ManARTS) Biobank, funded by the National Institute for Health Research (NREC 15/NW/0409), and the Allergy Unit of the Pneumonology Departments, Hospital Clinic, Barcelona, Spain. Patients had a well-documented history of an IgE-mediated reaction following ingestion of wheat- or gluten-containing foods, and evidence of sensitization to wheat and/or ω-5-gliadin determined by ImmunoCAP (ThermoFisher Scientific, Uppsala, Sweden) (>0.35kUsIgE/L) or ImmunoCAP ISAC (ThermoFisher Scientific, Uppsala, Sweden) I (>0.3 ISU) (Phadia-Thermo-Fisher).

### 2.3 Methods

#### 2.3.1 Preparation of Protein Fractions

The first-break white flour fraction was selected as it corresponds to the central part of the grain, with the lowest level of bran contamination, accounting for about 25% of the grain weight.^15^ Protein fractions were prepared as previously described^16^ (Figure S1). Briefly, flour was initially defatted by stirring with 10 volumes of hexane for 3 h at room temperature, filtered, and air-dried. The defatted flour (10 g) was extracted in 10 volumes of 0.5 M NaCl with stirring for 1 h before centrifugation at 5000*g* for 10 min at room temperature. The supernatant was collected, and the pellet re-extracted with 0.5 M NaCl; the resulting supernatants were pooled to give the albumin and globulin fraction (ALGL). The pellet was rinsed with deionized water for 2–3 times and then extracted in 10 volumes (to the initial flour weight) of 70% (v/v) aqueous ethanol by stirring for 1h at room temperature. After centrifugation at 5000*g* for 10 min at room temperature, the supernatant was removed, and the pellet was re-extracted in the same manner. The two supernatants were pooled to give the gliadin fraction. The pellet was then re-extracted twice in 10 volumes (to the initial flour weight) of 50% (v/v) aqueous propan-2-ol containing 60 mM dithiothreitol (DTT) and 1% (v/v) acetic acid to give the glutenin fraction. Protein was precipitated from the ALGL fraction by adding (NH_4_)_2_SO_4_ to 2.7 M and from the gliadin and glutenin fractions by adding 1.5 M NaCl, allowing to stand overnight at 4 °C. The resulting protein precipitates were collected by centrifugation at 5000*g* for 30 min at 4 °C, resuspended in 50 mL of either 0.5 M NaCl (ALGL fraction) or 0.1 M acetic acid (gliadin and glutenin fractions), and dialyzed overnight at 4 °C against 300 volumes of either 0.05 M NH_4_HCO_3_ (ALGL fraction) or water (gliadin and glutenin fraction) using 3 kDa molecular weight cutoff dialysis tubing, the buffer being changed at 2, 4, 6 h. The samples were then freeze-dried and stored at −20 °C.

Another set of wheat flour extracts was prepared as previously described.^17^ Briefly, nondefatted wheat flour was subjected to either a one-step extraction in 50% (v/v) propan-2-ol, 100 mM Tris–HCl, pH 7.5 containing 2 M urea and 60 mM DTT, a two-step extraction with 20 volumes of 60% (v/v) aqueous ethanol (two step 1) followed by 50% (v/v) aqueous propan-2-ol, 100 mM Tris–HCl, containing 2 M urea and 60 mM DTT (two step 2), with sonication at 60 °C for 10 min, or a one-step extraction with 50% (v/v) aqueous propan-2-ol, 100 mM Tris–HCl, containing 2 M urea and 60 mM DTT. A sample of gluten-free flour was also extracted using a one-step extraction procedure.

#### 2.3.2 Protein Determination

The protein content of the gliadin and glutenin fractions was determined using the Dumas combustion method, which measures the total nitrogen. Analysis was performed using a Leco combustion analyzer (Leco Corp., St. Paul, MN, USA) and was performed in duplicate. A conversion factor of 5.7 ^18^ was used to convert the nitrogen to the protein. The protein content of fractions was also determined in triplicate using the RC DC Lowry-based assay^19^ using bovine serum albumin (BSA) as the protein standard.

#### 2.3.3 Sodium Dodecyl-sulfate Polyacrylamide Gel Electrophoresis (SDS-PAGE)

Samples were resuspended in the corresponding extraction buffer to 1 mg protein/mL, then mixed at 1:1 (v/v) with LDS sample buffer containing 100 mM DTT, and heated at 90 °C for 10 min. The gel was then loaded with either protein marker or sample (∼10 μg/track), and gel electrophoresis was set at 200 V, 350 mA, and 100 W for 35 min. Gels were fixed in 50% (v/v) methanol and 10% (v/v) trichloroacetic acid for 1 h, rinsed for 5 min with deionized water, and stained with SimplyBlue. The gel was subsequently imaged using a GE Healthcare Typhoon Trio variable mode imager (GE Healthcare Lifesciences, Buckinghamshire, U.K.).

#### 2.3.4 Immunoblotting

SDS-PAGE separation was performed as in Section 2.3.3, except that SeeBlue prestained protein standards were used. After separation, the gel was soaked in transfer buffer (192 mM glycine, 25 mM Tris, and 20% (v/v) methanol) for 15 min. It was then laid on a prehydrated nitrocellulose membrane and sandwiched between presoaked filter papers in a Trans-blot semidry transfer cell (Bio-Rad, Hertfordshire, U.K.). Electroblotting was performed at 15 V, 300 A, and 100 W for either 25 min (one blot) or 35 min (two blots). The membrane was removed and washed twice for 10 min with phosphate-buffered saline (PBS), containing 0.05% (v/v) Tween 20 (PBST). The membrane was subsequently incubated with block buffer (PBST containing 5% (w/v) skim milk powder) for 1 h at room temperature. The blot was then rinsed 4 × 5 min in PBST before incubating with either primary animal antibodies (see Table S1) diluted at 1:5000 or 1:10,000 (v/v) or human serum diluted 1:10 (v/v) in blocking buffer and incubated overnight at 4 °C. After a further 4 × 5 min wash in PBST, the blot was then incubated with either alkaline phosphate (AP) conjugated secondary antibody specific for the relevant animal antibody or antihuman IgE antibody for 1 h at room temperature. The membrane was washed again with PBST for 4 × 5 min, before being incubated with NBT/BCIP solution for 10–15 min in the dark. Once the color had developed, the membrane was rinsed with deionized water, sandwiched between transparent films, and imaged using Bio-RAD Universal Hood II (Hertfordshire, U.K.). The densitometry analyses of the IgE immunoblots were processed in Image Lab (version 6.1), and the band intensity and relative molecular mass of bands were exported to a .csv file format and further analyzed using GraphPad Prism (version 9.1.2).

#### 2.3.5 Sample Preparation for Liquid Chromatography Tandem Mass Spectrometry (LC-MS/MS) Analysis

Synthetic peptides P1, P2, P6, and P7 (as listed in Table 1) were prepared in either 5% (v/v) aqueous acetonitrile or digested gluten-free flour matrix, and the unlabeled light peptides were diluted to produce calibrants at 500, 250, 100, 50, 25, 10, 5, 2, 1, and 0.1 fmol/μL. A mix of isotopically labeled peptides was prepared by combining each peptide stock solution and was added to each sample or calibrant, to give a final concentration of 25 fmol/μL for each peptide.

**Table 1:** Target Peptides for Multiple Reaction Monitoring (MRM) in LC-MS/MS^a^

peptide sequence (charge state)	uniprot accession ID	protein type	monitoring window (min)	precursor (*m*/*z*)	transition (*m*/*z*)
P1: RPQQPYPQPQPQ**Y** ^23–26^ (2+)	P02863	α-gliadin	10.50–11.50	813.9048	***[y3] - 407.1925***
	P04721				[y5] - 632.3039
	P04722				[b8] - 995.5057
					[b10] - 1220.6171
P2: QPFPQPQLP **Y** (2+)	P02863	α-gliadin	15.20–16.10	607.8139	[b5] - 598.2984
	P04722				***[b8] - 936.4938***
	P04724				[b7] - 823.409
	P18573				
P3: IPPHCSTTIAPF (2+)	P04725	α-gliadin	13.50–14.70	670.8370	***[b10] - 1078.5350***
	P04727				[b9] - 1007.4979
					[b8] - 894.4138
P4: ASIVAGISGQ (2+)	B6UKP3	γ-gliadin	11.90–13.00	451.7507	***[b7] - 612.3715***
					[y5] - 461.2354
					[y6] - 532.2726
P5: ASIVAGIGGQ (+)	P08453	γ-gliadin	12.00–13.00	872.4836	***[y5] - 431.2249***
					[y6] - 502.2620
					[b7] - 612.3715
P6: GIIQPQQPAQ **L** (2+)	P08453	γ-gliadin	13.10–13.80	596.8379	***[y7] - 781.4203***
	P21292				[b7] - 765.4254
					[b4] - 428.2504
P7: VQQQIPVVQPSI**L (2+)** ^26,27^	P10386	LMW-GS	16.00–16.80	724.9272	***[b5] - 597.3355***
					[y4] - 429.2708
					[y8] - 852.5189
					[y5] - 557.3293
P8: GVGTGVGAY (+)	P10386	LMW-GS	10.20–11.50	780.3886	[y4] - 409.2082
	P04729				***[b6] - 471.2562***
					[b8] - 599.3148
					[y7] - 624.2988
P9: GQCVSQPQQQSQQQL (2+)	P10386	LMW-GS	9.20–10.40	872.4076	***[b6] - 660.2770***
	P04730				[y9] - 1084.5382
	P04729				[b4] - 445.1864

a All cysteines were alkylated. Isotopically labeled amino acids in peptides P1, P2, P6, and P7 are indicated in **bold**. The position of coeliac toxic motifs is underlined: PFPQPQLPY^20^ GIQPQQPAQL.^21,22^ Quantifier ions are shown in bold italic. Precursor and transition (*m*/*z*) values are given for the light peptides.

Osborne fractions were prepared for LC-MS/MS analysis as summarized in Figure S2. The ALGL and gliadin fractions were prepared in duplicate in 0.5 M NaCl and 70% (v/v) aqueous ethanol, respectively, at a concentration of 2 mg protein/mL, while the glutenin fraction was prepared in duplicate in 50% (v/v) aqueous propan-2-ol, 60 mM DTT, and 1% (v/v) acetic acid at 1.5 mg protein/mL. DTT was added to the ALGL and gliadin fractions to a final concentration of 60 mM, and all samples were incubated at 60 °C for 10 min. All samples were then alkylated by adding iodoacetamide to a final concentration of 120 mM, and reduced bakers’ yeast enolase, which was used as an internal protein standard, was added to a final concentration of 10 μg/mL before incubating at ambient temperature in the dark for 30 min. The alkylated samples were diluted to 250 μg of protein/mL in chymotrypsin digestion buffer (100 mM Tris–HCl, containing 10 mM CaCl_2_) with the addition of RapiGest SF to a final concentration of 0.1% (w/v). Chymotrypsin, prepared in chymotrypsin digestion buffer, was added at a protease-to-protein ratio of 1:100 (w/w), and the samples were digested overnight at 37 °C. Digestion was quenched by adding formic acid to a final concentration of 0.5% (v/v), and the supernatants were collected after centrifugation. Samples were then centrifuged at 10,000*g* for 10 min, and the supernatants were removed and applied to SepPak C18 columns, which had been prewashed with acetonitrile and conditioned with 0.1% (v/v) aqueous FA. Bound peptides were eluted first with 20% (v/v) and then 80% (v/v) acetonitrile. Eluates were pooled and concentrated by vacuum centrifuge before adding the mixed isotopically labeled peptides to a final concentration of 25 fmol/μL.

#### 2.3.6 LC–MS/MS Analysis

Targeted mass spectrometry analysis was performed using an Xevo TQ-S triple quadrupole mass spectrometer (Waters Corporation, Manchester, U.K.) coupled with an ACQUITY UPLC M-class system (Waters Corporation, Milford, MA, USA). The system was equipped with a Symmetry C18 100 Å, 5 μm, 300 μm × 50 mm trap column (Waters Corporation, Milford, MA, USA) and an ionKey Peptide BEH C18 300 Å, 1.7 μm, 150 μm × 100 mm analytical column (Waters Corporation, Milford, MA, USA). The instrument operated in trap-and-elute mode, with a mobile phase A consisting of water containing 0.1% (v/v) FA and a mobile phase B consisting of acetonitrile with 0.1% (v/v) FA. The gradient started with a flow rate of 15 μL/min of 1% (v/v) mobile phase B for the first 3 min while trapping and diverted to waste before valve switching and a reduction in flow rate of 2 μL/min across the analytical column. The gradient was programmed as follows: 0 min at 5% (v/v) mobile phase B, 2 min at 5% (v/v) mobile phase B, 14 min at 30% (v/v) mobile phase B, 15 min at 40% (v/v) mobile phase B, 17–20 min at 65% (v/v) mobile phase B, and 22–26 min at 5% (v/v) mobile phase B. The monitored time windows for each peptide marker are shown in Table 1. Samples were analyzed in triplicate with an injection volume of 3 μL.

#### 2.3.7 Data Processing and Statistical Analysis

The raw data generated from Xevo TQ-S were directly processed through Skyline (version 21.2) and manually validated. Data were then exported from Skyline as .csv files for further analysis. Data were retained when the intensity of the raw signal was three times the signal-to-noise ratio (S/N). For each peptide, the most intense transition was selected as the quantifier. In samples analyzed using heavy isotopically labeled peptides, the peak area ratios of the endogenous light peptide reporter to the corresponding heavy-labeled peptide standard were calculated. The ratio was then multiplied by the concentration of the heavy spike, taking into account the dilution during sample preparation used to calculate the peptide concentration in the unknown sample. Statistical analysis, including analysis of variance (ANOVA), was performed by using GraphPad Prism (version 9.1.2). Principal component analysis (PCA) was conducted in MetaboAnalyst 5.0.^28^ Standard curves for each peptide were generated in GraphPad Prism. The limits of detection (LOD) and quantitation (LOQ) of each peptide were calculated using eqs 1 and 2, respectively:^29^

1 $$\mathrm{LOD}=\mathrm{LLOD}+3S_{y/x}$$

2 LOQ=3LOD

where *S*_*y*/*x*_ represents the standard deviation of the residuals in the *y*-axis direction. The lower limit of detection (LLOD) was the lowest concentration point at which the peptide transition peak height was higher than three times the S/N ratio of the blank samples (either 5% acetonitrile or gluten-free flour matrix extract).

The on-column peptide quantification was carried out by first calculating the light-to-heavy peak area ratio (PAR) and then multiplying the PAR by the amount of isotopically labeled peptide that was injected onto the column (25 fmol) for each sample.

Peptide-to-protein conversion factors were calculated by using two different approaches (Figure 1).

**Figure 1:**
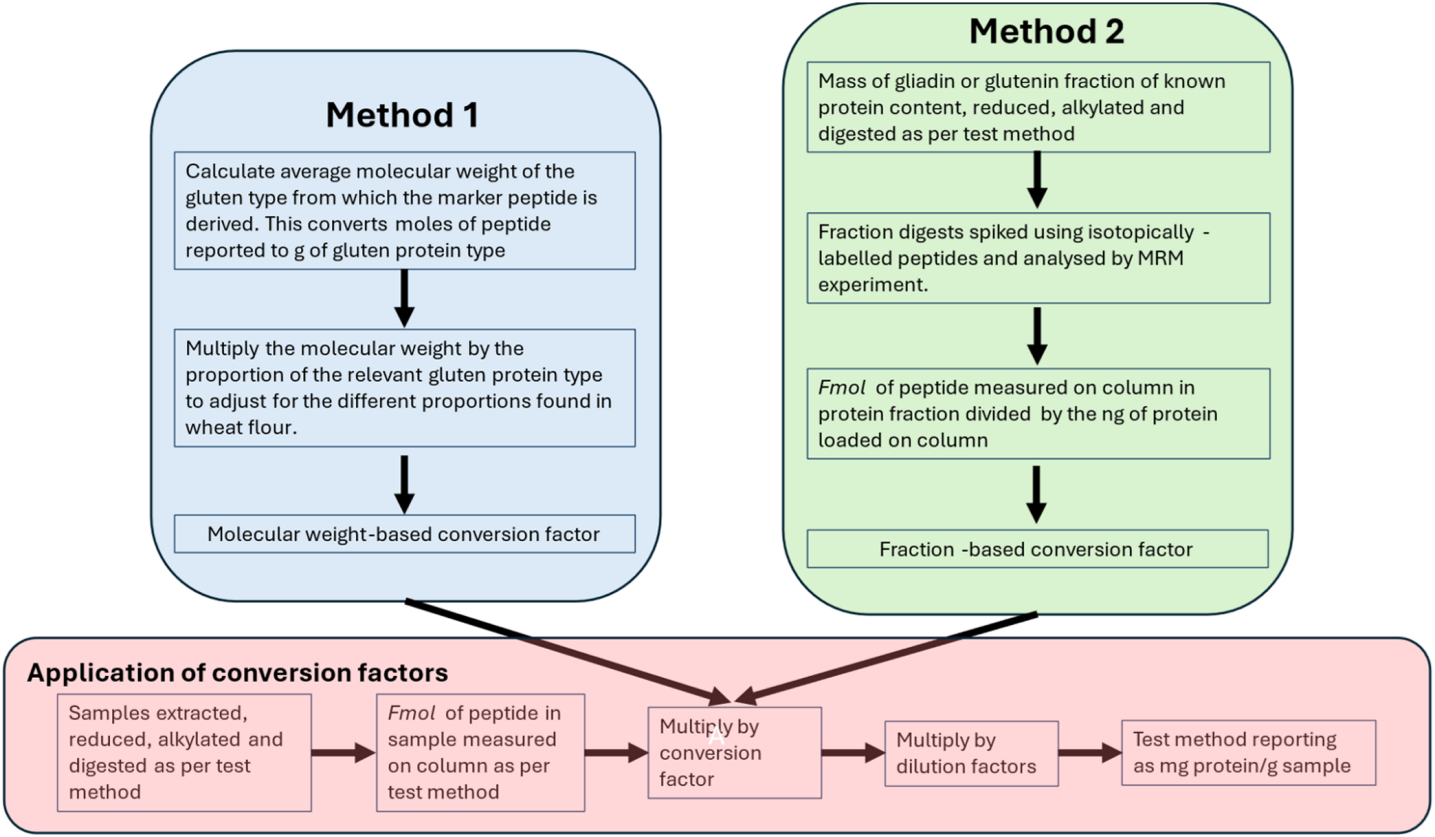
Flowchart describing the two different methods used to calculate peptide-to-protein conversion factors and their application in a mass spectrometry method for determining gluten protein.

Method 1: The average molecular weights of the gluten protein type (i.e., α- or γ-gliadin or LMW-GS) from which the peptide marker originated was calculated using relevant protein sequences in the GluPro v6.1 database.^30^ These were 30,367.09 Da for the α-gliadin peptide P1 (RPQQPYPQPQPQY), 30,569.85 Da for the γ-gliadin peptide P6 (GIIQPQQPAQL), and 31,622.33 Da for the LMW-GS peptide P7 (VQQQIPVVQPSIL). This was used to convert from moles of peptide detected to mass (g) of the protein type. The proportions of the different gluten protein types (based on data from Schalk et al.^31^ as follows: α-gliadin 33.5%; γ-gliadin 21.6%; and LMW-GS for 24.9%) were then taken into account to give the final conversion of peptide to gluten protein.

Method 2: Isotopically labeled peptide markers were spiked into reduced, alkylated, and chymotrypsin-digested gliadin and glutenin fractions of known protein concentration, and the peptide concentrations were quantified by MRM analysis. The fraction-based conversion factors were then calculated using eq 3 with the units of mg protein mole peptide^–1^.

3 $$conversion\;factor=\frac{mg\;protein\;p\mathrm{er}\;fraction\;\left(RC\;DC\;\mathrm{or Dumas combustion}\right)}{dilution\;factor\times fmol\;peptide\;measured\;on\;column}$$

The conversion factors were applied to the amount of peptide marker determined in the test samples, taking into account the dilution factors, as described in eq 4 (see Figure S2 for detailed workflow).

4 proteinpergflour=(fmolpeptidemeasuredoncolumn×conversionfactor×dilutionfactor)/mgflour

## 3 Results

### 3.1 Immunoblotting of Wheat Protein Fractions with Animal Antibodies

It is crucial that fractions used to derive conversion factors for mass spectrometry have a representative protein composition. Consequently, the initial focus was on characterizing the protein compositions of the wheat flour fractions. First, protein fractions were analyzed using SDS-PAGE and immunoblotting with a range of specific animal antibody preparations (Figure 2 and Table S1) and HPLC (Supplementary Results S2, Figures S7–S10).

**Figure 2:**
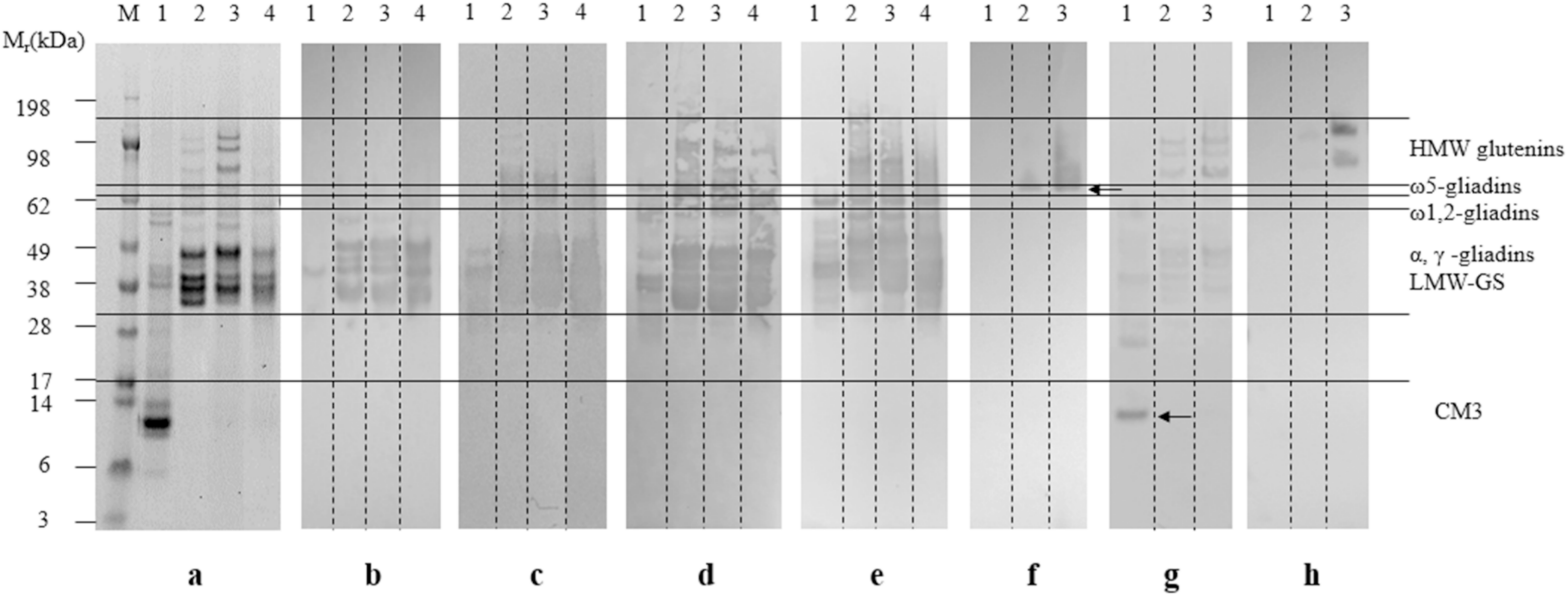
SDS-PAGE and immunoblotting analyses of the Osborne fractions of wheat. (a) Gel was stained for protein. M: molecular weight markers; tracks 1—ALGLs; 2—gliadins; 3—glutenins; 4—PWG-gliadin. (b–g) Immunoblots of fractions were developed with different antibody preparations. Antibody preparations were R5 (b), G12 (c), IFRN 0065 (d), IFRN 0610 (e), ONT18A5 (f), CM3 (g), and IFRN 1602 (h). The positions of ω5-gliadins and CM3 were indicated by arrows. Approximately 10 μg protein was loaded per lane, apart from the anti-CM3 blot (g), where ∼7 μg of protein was loaded per lane.

The ALGL fraction profile comprised a mixture of polypeptides of *M*_r_ ∼ 60 kDa, accompanied by a group of polypeptides of *M*_r_ 38–45 kDa and a strongly staining band of *M*_r_ ∼ 14 kDa. The latter is consistent with the molecular weight of α-amylase/trypsin inhibitors (ATIs),^32^ and this identity was confirmed by immunoblotting with an antibody to the ATI CM3. In addition to the *M*_r_ ∼ 14 kDa band, the antibody preparation recognized two additional bands of *M*_r_ ∼ 25 and ∼38 kDa, which may represent oligomeric forms, as previously observed in purified protein fractions.^33^ The ALGL fraction was also analyzed by immunoblotting with four antigluten antibody preparations, all of which recognized polypeptides, specifically bands of *M*_r_ ∼ 43 kDa for G12, *M*_r_ 35–60 kDa for R5, and *M*_r_ 28–62 kDa for IFRN 0065 and IFRN 0610 (Figure 2b–e). These data indicate the presence of gliadins in the salt-soluble ALGL fraction, but there was no evidence of ω5-gliadins or high-molecular-weight subunits of glutenin (HMW-GS) based on immunoblots of ONT18A5 and IFRN 1602, respectively (Figure 2f,h).

The gliadin fraction extracted with aqueous ethanol had abundant bands of *M*_r_ 32–55 kDa, consistent with their being monomeric α- and γ-type gliadins^34,35^ and was very similar to the PWG-gliadin fraction with very faint bands at *M*_r_ ∼ 60 kDa assigned to ω-gliadins (Figure 2a tracks 2 and 4, respectively). These results are consistent with the profile of the PWG-gliadin reported previously.^11^ Several polypeptides of *M*_r_ ∼ 62 kDa, which probably correspond to ω5-gliadins,^36^ were recognized by the ONT18A5 antibody (Figure 2f). The antibody preparations G12, R5, IFRN 0065, and IFRN 0610 also recognized substantial portions of the gliadins, together with some alcohol-soluble LMW-GS. Faintly staining bands were observed above *M*_r_ ∼ 70 kDa, which were recognized by IFRN 1602 (Figure 2h), showing that traces of HMW-GS were present in the gliadin fraction. The anti-CM3 antibody also recognized polypeptides at *M*_r_ ∼ 30 and 48 kDa, but no binding to the CM3 band at *M*_r_ 14 kDa was observed.

Analysis of the reduced glutenin fraction revealed distinct bands in the range of *M*_r_ 80–120 kDa, corresponding to HMW-GS, together with bands of *M*_r_ 38–49 kDa, which corresponded to LMW-GS.^37^ The antibodies G12, R5, IFRN 0065, and IFRN 0610 showed binding patterns similar to those observed for the gliadin fraction. This reflects the presence of sequences recognized by IFRN 0065 and 0610 in both gliadins and LMW-GS. The ONT18A5 antibody recognized multiple bands ranging from *M*_r_ ∼ 62 to 120 kDa (Figure 2f), indicating that some ω5-gliadins may be associated with the HMW-GS.^38^ As with the gliadin fraction, the anti-CM3 antibody preparation recognized proteins in the glutenin fraction of *M*_r_ ∼ 30–55 kDa while IFRN 1602 recognizing higher molecular weight polypeptides corresponding to the HMW-GS (Figure 2h).

### 3.2 Immunoblotting of Protein Fractions with IgE from Serum of Wheat Allergic Patients

The IgE reactivity of the Osborne fractions was subsequently investigated using a serum panel from patients with IgE-mediated wheat allergy to ensure their clinical relevance, as has been done for the development of allergen test materials^39^ (Figures 3, S4, and S5). Most of the patients had experienced anaphylactic reactions, while some also suffered from acute urticaria and angioedema (Table S2). Only two patients, Q and H, displayed IgE binding to the ALGL fraction, with faint binding observed to polypeptides of *M*_r_ ∼ 40 and 15 kDa, while patient H demonstrated strong binding to a polypeptide of *M*_r_ ∼ 25 kDa accompanied by faint recognition of the *M*_r_ ∼ 15 kDa polypeptide. The latter polypeptide may correspond to an ATI (Figures 3a and S5). By contrast, strong IgE binding was observed in the gliadin and glutenin fractions, with similar but distinct binding patterns of binding to bands at *M*_r_ ∼ 70, 55, and 40 kDa, which may correspond to ω5-gliadins, α-, γ-gliadin, and LMW-GS, respectively. Interestingly, none of the patients reacted only to ω5-gliadins while 8 patients reacted only to α-, γ-gliadin and LMW-GS (A, D, E, I, L, N, R, V) and 11 reacted to ω5-gliadins and other gliadins (B, C, G, I, J, K, M, P, S, T, U). Although the ImmunoCAP results indicated that all patients, except patient E, were sensitized to ω5-gliadin, only 12 patients showed IgE binding to ω5-gliadin on immunoblots, which may be due to its lower sensitivity compared to the ImmunoCAP (Table S2 and Figure S5). Densitometric analysis of all of the IgE immunoblots showed binding to the *M*_r_ 70, 55, and 40 kDa polypeptides in all patients (Figure 3b).

**Figure 3:**
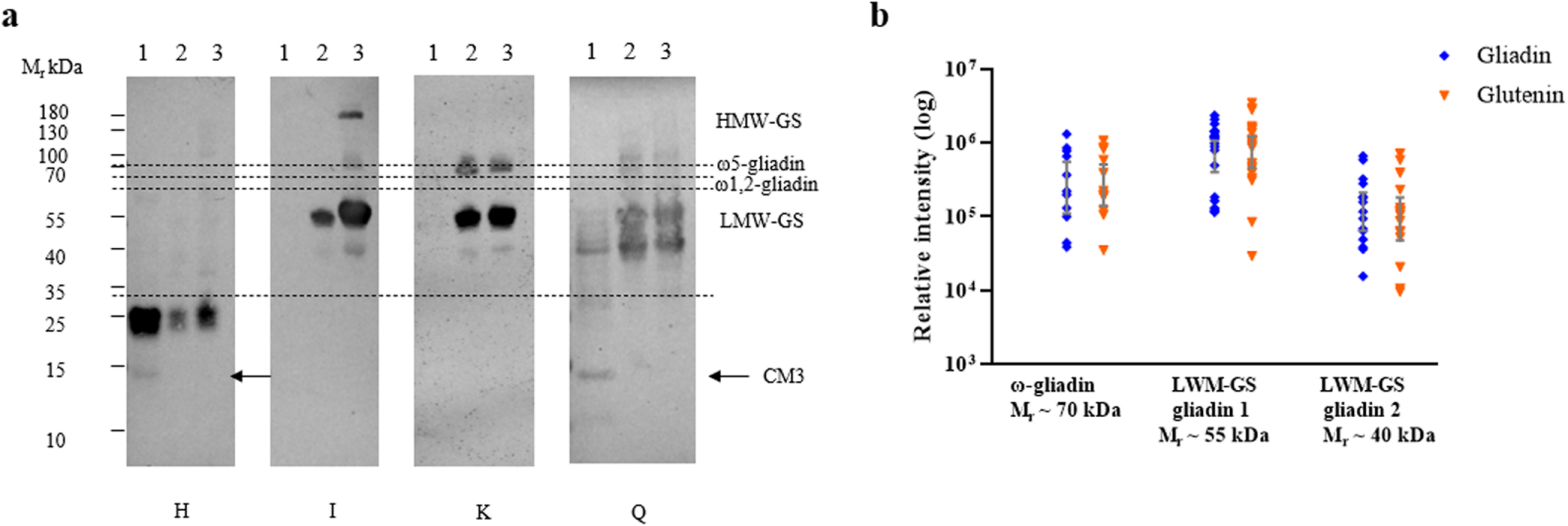
IgE reactivity of the Osborne fractions. (a) Examples of blots with patient sera showing different patterns of reactivity—patient H, there were specific recognition at *M*_r_ ∼ 25 and 14 kDa; patient I—recognition of gliadins, glutenins, and HMW-GS; patient K—recognitions of gliadins and glutenins; patient Q—recognition of ALGL fraction, gliadins, and glutenins. (b) Densitometry analysis of IgE-reactive bands of *M*_r_ ∼ 70, 55, and 40 kDa in the gliadin and glutenin fraction (see also Figures S4 and S5 for data for all patients).

### 3.3 Analysis of Wheat Flour Fractions Using Prolamin-Specific Peptide Targets by Multiple Reaction Monitoring Mass Spectrometry

A suite of wheat-specific peptide markers (Table 1) was identified from discovery proteomics data developed using a chymotryptic workflow. These peptides were selected based on a range of criteria, including specificity for different gluten protein fractions, abundance, and immunoreactivity.^40^ Three peptides were chosen to represent α-gliadins, three for γ-gliadins, and three for LMW-GS, two of which contain celiac toxic motifs (QPFPQPQLPY for α-gliadins and GIIQPQQPAQL for γ-gliadins). First, their performance was evaluated using multiple reaction monitoring (MRM) mass spectrometry analysis of the Osborne fractions to select the best-performing peptides to be synthesized as isotopically labeled peptide standards. All but two of the peptides showed stable fragment ion transitions across the Osborne fractions (Figure S3), although their proportions varied (Figure 4 and Table S3). The exceptions were the α-gliadin peptide QPFPQPQLPY, which was detected only in the ALGL fraction, indicating greater solubility of the protein(s) carrying this CD motif in dilute salt solution compared to the aqueous alcohols typically used to solubilize gliadins. The other exception was the LMW-GS peptide, GVGTGVGAY, which was not detected in the ALGL fraction but was present in both the gliadin and glutenin fractions.

**Figure 4:**
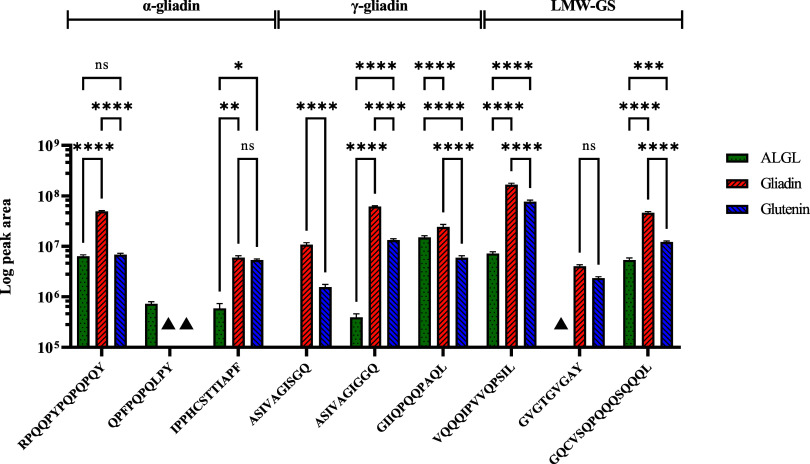
Targeted LC-MS analysis of the Osborne fractions using candidate peptide markers. The peak area for each peptide was determined by the quantification ion (Table 1). The average total peak area, standard deviation (SD), and % coefficient of variation (CV) are shown in Table S3. ▲—no detectable peptide. ns = not significant (*p* > 0.05), * = 0.05 > *p* > 0.01, ** = 0.01 > *p* > 0.001, *** = 0.001> *p* > 0.0001, and **** = *p* < 0.0001.

The α-gliadin peptide RPQQPYPQPQPQY, the γ-gliadin peptides ASIVAGISGQ and ASIVAGIGGQ, and the LMW-GS peptides VQQQIPVVQPSIL and GQCVSQPQQQSQQQL were all more abundant in the gliadin fraction compared to the ALGL and glutenin fractions. By contrast, the levels of the α-gliadin peptide IPPHCSTTIAPF did not vary significantly between all three fractions, while the γ-gliadin peptide GIIQPQQPAQL was more abundant in the ALGL and gliadin fractions compared with the glutenin fractions. These observations can be explained by the fact that although they were chosen to be “class-specific” by screening using the GluPro V6 database,^30^ the peptide sequences may actually occur within different gliadin and glutenin proteins, and/or there was cross-contamination of the different fractions.

### 3.4 Peptide to Protein Conversion Factors for the Quantification of Prolamins by Targeted Mass Spectrometry Using Multiple Reaction Monitoring

Based on this preliminary analysis, four peptides were selected for quantitative analysis using isotopically labeled standards to derive peptide-to-protein conversion factors. The most abundant transition ion was chosen as the quantifier ion for each peptide: y3 for RPQQPYPQPQPQY, b8 for QPFPQPQLPY, y7 for GIIQPQQPAQL, and b5 for VQQQIPVVQPSIL. The ion ratio for the corresponding quantifier ion of each peptide was monitored to ensure consistency throughout the quantification (Table S4). In order to take account of matrix effects in absolute quantification, the peptide markers were also spiked into either solvent alone or a gluten-free flour matrix extract to generate isotopic dilution (SID) curves; the distribution of the transition ions for each peptide marker is shown in Figure 5. All four peptides exhibited good linearity with *r* ^2^ > 0.99. The limits of detection (LODs) ranged from 1 to 7 fmol of peptides on column, while the limits of quantification (LOQs) were between 5 and 21 fmol. The γ-gliadin peptide GIIQPQQPAQL had the lowest LOD and LOQ, at 1.9 and 5.8 fmol on column, respectively (Table S5). We observed that when using the gluten-free matrix, the LOQs were generally higher compared to those in buffer, except for peptide VQQQIPVVQPSIL, indicating minimal matrix effects on the quantification performance.

**Figure 5:**
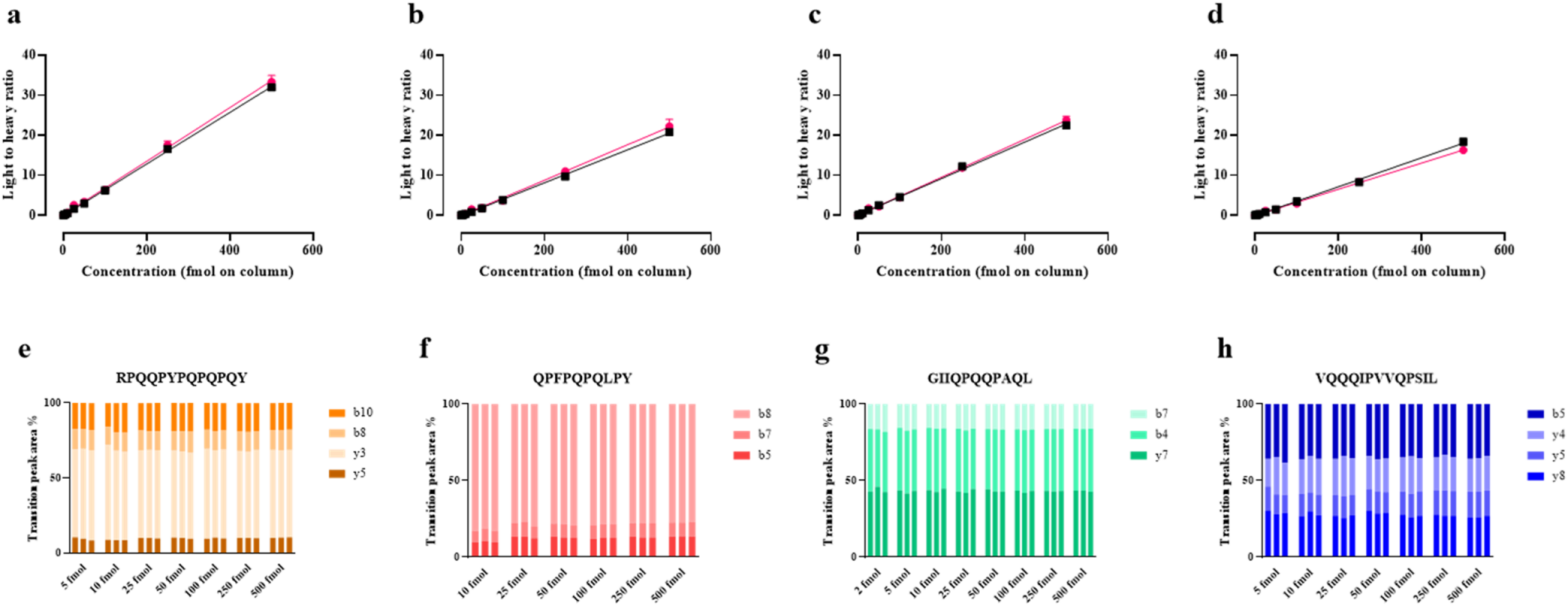
Serial isotopic dilution curves and corresponding transitions for heavy-labeled peptide markers analyzed using the MRM method. Calibration curves (a–d) were created using peptide concentrations of 0–500 fmol/μL and using samples with qualifying signal-to-noise ratios and ratios of quantifying peak area to total peak area. Transition ratios for each peptide in buffer are shown in panels (e)–(h) and in the gluten-free flour matrix in Figure S6. Peptides were prepared in either 5% (v/v) aqueous acetonitrile (■) or gluten-free flour matrix extract (pink solid circle) as follows: (a, e) RPQQPYPQPQPQY; (b, f) QPFPQPQLPY; (c, g) GIIQPQQPAQL; and (d, h) VQQQIPVVQPSIL. Samples were analyzed in triplicate.

In order to establish the fraction-based conversion factors (Method 2), the isotopically labeled peptide markers were spiked into the reduced, alkylated, and digested Osborne fractions, and the yield of marker peptide per mg of protein fraction was calculated (Table 2). This analysis confirmed that the peptide QPFPQPQLPY was only present at very low levels in the fractions, even in the ALGL. The α-gliadin peptide RPQQPYPQPQPQY was most abundant in the gliadin fraction, while the γ-gliadin peptide GIIQPQQPAQL was present at almost equal abundance in the ALGL and gliadin fractions. The LMW-GS peptide VQQQIPVVQPSIL was more abundant, in the gliadin and glutenin fractions. The results of this analysis, together with the protein content of the gliadin and glutenin fractions (Table 3), were then used to derive a set of conversion factors (Method 2) for each peptide apart from peptide P2 (QPFPQPQLPY), which was of too low an abundance to be used further (Table 4). Conversion factors based on the ALGL fraction were not used since gluten proteins were a minor constituent, and no protein determination using total nitrogen was available (Table S7). The slight differences in protein content determined by different assays had minimal impact on the conversion factors. For example, the conversion factors for the α-gliadin peptide RPQQPYPQPQPQY determined using the gliadin fraction were either 1.55 ng protein/fmol peptide (gliadin protein determined using the RC DC assay) or 1.84 ng protein/fmol peptide (gliadin protein determined using the Dumas combustion assay). Given that the Dumas combustion assay is a standard reference assay for nitrogen with better reliability and reproducibility, the Dumas-derived conversion factors were used for subsequent calculations.^41,42^

**Table 2:** Detection of Gluten Peptide Markers in Osborne Fractions

		fmol peptide on column		
peptide marker	protein type	ALGL fraction	gliadin fraction	glutenin fraction
P1: RPQQPYPQPQPQY	α-gliadin	24.2 ± 2.0	109.8 ± 19.2	24.1 ± 1.2
P2: QPFPQPQLPY	α-gliadin	<LOQ	<LOQ	<LOQ
P6: GIIQPQQPAQL	γ-gliadin	47.3 ± 5.1	50.3 ± 12.6	19.4 ± 1.8
P7: VQQQIPVVQPSIL	LMW-GS	27.2 ± 4.7	424.1 ± 78.4	336.6 ± 21.2

**Table 3:** Protein Content of the Osborne Fraction by RC DC Assay and the Dumas Combustion Assay (*N* × 5.7)^a^

	protein % (w/w)	
Osborne fraction	extracted protein content by RC DC assay	extracted protein content by Dumas combustion
gliadin fraction	57.5 ± 0.002	69.2, 67.4
glutenin fraction	91.9 ± 0.03	88.0, 88.9

a Mean ± SD, RC DC assay was measured in triplicate, while the Dumas combustion was measured in duplicate.

**Table 4:** Conversion Factors Generated Using Method 2: Gliadin and Glutenin Fraction Protein Was Quantified Using Either the RC DC Assay or the Dumas Combustion Assay^a^

		gliadin fraction (ng gluten protein/fmol peptide)		glutenin fraction (ng gluten protein/fmol peptide)	
peptide marker	protein type	RC DC	Dumas combustion	RC DC	Dumas combustion
P1: RPQQPYPQPQPQY	α-gliadin	1.55	1.84	13.72	13.18
P6: GIIQPQQPAQL	γ-gliadin	3.38	4.01	17.03	16.36
P7: VQQQIPVVQPSIL	LMW-GS	0.40	0.48	0.98	0.95

a Calculated from eq 3.

The conversion factors varied significantly between the gliadin and glutenin fractions. Specifically, the conversion factor for the γ-gliadin peptide GIIQPQQPAQL was 4.01 ng of protein/fmol of peptide using the gliadin fraction and 16.36 ng of gluten protein/fmol of peptide using the glutenin fraction. For the LMW-GS peptide VQQQIPVVQPSI, the conversion factors were lower, at 0.48 ng of gluten protein/fmol of peptide for the gliadin fraction and 0.95 ng of gluten protein/fmol of peptide for the glutenin fraction, respectively.

### 3.5 Application of Mass Spectrometry Analysis to Quantification of Gluten Proteins in Wheat Flour

A set of previously characterized wheat flour gluten protein extracts prepared using either a simple single-step or two-step extraction procedure^17^ were then subjected to the reduction, alkylation, and digestion protocol and analyzed using the quantifier ion ratios for peptide markers P1, P6, and P7 (Table S6). These were chosen since the extraction procedures were shown to maximize recovery of gluten protein and for which protein levels were available.^17^ The single-step procedure employed a combination of propan-2-ol, urea, and DTT to maximize extraction of gliadins and glutenins, while the two-step procedure utilized a first step extraction in 60% ethanol (as indicated in the CODEX standard) followed by a second step to reextract the remaining pellet in the propan-2-ol, urea, and DTT buffer. Test results were converted to protein using two different methods (Figure 1).

#### 3.5.1 Conversion Factor Method 1

The first approach used was to convert the peptide mass to protein mass using the average molecular weight of the parent gluten protein type, based on sequences in the GluPro v 6.1 database^30^ and adjusting the factor to account for the proportion of that protein type in wheat gluten (Figure 1). Using this method, the quantified gluten protein content ranged from 0.4 ± 0.05 mg/g flour to 11.7 ± 2.9 mg/g flour. This significantly underestimated the protein level by approximately two orders of magnitude compared to the conventional total protein measurement,^17^ which indicated the extracts comprised around 150 mg protein/g flour for both one-step and combined two-step extractions (Table 5). The highest protein quantity for one-step extraction was 7.0 ± 0.5 mg of gluten/g of flour, quantified by peptide VQQQIPVVQPSIL, and that for the combined two-step extraction was 11.7 ± 2.9 mg of gluten/g of flour, quantified by peptide RPQQPYPQPQY. This approach assumes complete digestion of a protein to release the peptides and their stability through the digestion, and it may be that this was incomplete, or the abundance of the peptide marker sequence in the gluten proteins was low, leading to the low estimates of gluten content.

**Table 5:** Quantification of Gluten in Wheat Extracts Using Conversion Method 1^a^

	fmol peptide on column				mg gluten protein/g flour			
	extraction method				extraction method			
peptide marker	one step	two step 1	two step 2	combined two steps	one step	two step 1	two step 2	combined two steps
P1: RPQQPYPQPQPQY	24.6 ± 1.3	114.3 ± 6.3	349.3 ± 104.2	463.6 ± 99.0	1.2 ± 0.1	2.9 ± 0.2	8.8 ± 2.9	11.7 ± 2.9
P6: GIIQPQQPAQL	19.9 ± 2.0	30.3 ± 2.2	44.6 ± 13.7	74.9 ± 11.5	0.5 ± 0.1	0.4 ± 0.05	0.5 ± 0.2	0.9 ± 0.2
P7: VQQQIPVVQPSIL	331.0 ± 20.8	258.6 ± 10.6	156.3 ± 41.3	415.0 ± 32.5	7.0 ± 0.5	2.7 ± 0.1	1.7 ± 0.5	4.4 ± 0.5
extracted protein content determined using the RC DC protein assay (from Daly et al.^17^) (±SD, *n* = 3)					151.8 ± 43.6	80.8 ± 1.8	73.4 ± 18	154.2 ± 16.4
% recovery of flour protein determined using the Dumas protein assay (from Daly et al.^17^) (±SD, *n* = 3)					122.5 ± 35.1	65.2 ± 1.4	59.2 ± 14.5	124.4 ± 13.2

a Results are given (±SD, *n* = 6).

#### 3.5.2 Conversion Factor Method 2

The second approach involved applying the conversion factors developed by using the gliadin and glutenin fractions. The same sample preparation protocol was used for the flour samples as had been used for analysis of the gluten protein fractions from which conversion factors were derived to ensure comparability. Applying factors calculated using Method 2 provided a more accurate quantification of gluten protein when using the conversion factor for the gluten protein type from which the peptide marker was derived. For peptide P1 (RPQQPYPQPQPQY), using the gliadin conversion factor gave 25.1 ± 1.5 mg gluten/g flour for the one-step extraction and 236.9 ± 58.4 mg gluten/g flour for the combined two-step extraction, whereas the glutenin conversion factor overestimated the protein amount by 5 to 10 times. Although peptide P6 (GIIQPQQPAQL) is a gliadin-derived peptide marker, using the glutenin conversion factor provided more accurate quantification, yielding 181.5 ± 19.8 mg gluten/g flour for the one-step extraction and 341.0 ± 69.0 mg gluten/g flour for the combined two-step extraction. For the LMW-GS peptide P7 (VQQQIPVVQPSIL), as expected, the glutenin conversion factor gave more accurate quantification than the gliadin conversion factor, with results of 174.7 ± 12.0 mg of gluten/g of flour and 109.5 ± 12.3 mg of gluten/g of flour for one-step and two-step extractions, respectively. In summary, the LMW-GS peptide VQQQIPVVQPSIL with glutenin conversion factors gave the most accurate quantification. An alternative approach, using the gliadin conversion factor for the first step of the two-step extraction protocol (which largely extracts gliadins) and the glutenin conversion factor for the second step of the two-step protocol (which extracts any remaining protein), gave only a slight improvement for P6, giving 236.7 ± 65.5 mg of gluten protein/g of flour compared with 83.5 ± 16.9 (gliadin conversion factor only) or 341.0 ± 69.0 (glutenin conversion factor only) (Table 6).

**Table 6:** Quantification of Wheat Gluten in Flour Extracts Using Conversion Method 2^a^

	mg gluten protein/g flour gliadin conversion factor				mg gluten protein/g flour glutenin conversion factor			
	extraction method				extraction method			
peptide marker	one step	two step 1	two step 2	combined two steps	one step	two step 1	two step 2	combined two steps
P1: RPQQPYPQPQPQY	25.1 ± 1.5	58.4 ± 3.5	178.5 ± 58.3	236.9 ± 58.4	180.1 ± 10.8	418.4 ± 25.4	1278.9 ± 417.9	1697.3 ± 418.7
P6:GIIQPQQPAQL	44.4 ± 4.8	33.8 ± 2.7	49.7 ± 16.7	83.5 ± 16.9	181.5 ± 19.8	138.1 ± 11.1	202.9 ± 68.1	341.0 ± 69.0
P7: VQQQIPVVQPSIL	88.3 ± 6.1	34.5 ± 1.5	20.8 ± 6.0	55.3 ± 6.3	174.7 ± 12.0	68.3 ± 3.1	41.3 ± 11.9	109.5 ± 12.3

a Results are given (±SD, *n* = 6).

## 4 Discussion

A set of well-characterized Osborne fractions was prepared from wheat flour to allow the performance of a set of candidate peptide biomarkers for gluten determination by MS to be evaluated. The fractions were also used as the basis for a protocol to derive factors to convert from peptide to protein. The protein profiles of each fraction determined by SDS-PAGE, immunoblotting analysis, and reverse phase-high-performance liquid chromatography (RP-HPLC) were consistent with those described in previously published studies^16,31,43^ and demonstrated that they were representative of the repertoire of gluten proteins present in wheat flour. This showed the presence of glutenin components, such as traces of HMW subunits of glutenin in the classical gliadin fraction extracted with 70% ethanol as well as the glutenin fraction extracted with 50% (v/v) aqueous propan-2-ol containing 60 mM DTT and 1% (v/v) acetic acid. Notably, the ω5-gliadins, which are monomeric proteins, were observed in the glutenin fraction, which is consistent with reports that certain ω-gliadins can associate with HMW-GS through noncovalent bonds, particularly hydrogen bonds.^38,44^ Finally, IgE immunoblotting verified the clinical relevance of the protein profiles regarding IgE-mediated wheat allergy, demonstrating the serum IgE reactivity to both the gliadin and glutenin fractions and complementing the analysis of celiac toxic motifs undertaken using two of the selected peptide markers. One patient with baker’s asthma showed faint serum IgE reactivity to a polypeptide likely to be an ATI together with strong IgE reactivity to a *M*_r_ ∼ 25 kDa polypeptide, which was not observed on the stained SDS-PAGE gel. This may correspond to a thiol reductase homologue, named Tri a 27, one of the critical allergens for bakers’ asthma.^45^

These well-characterized fractions were then used to evaluate a suite of candidate peptide markers for gluten quantification by MS using a chymotryptic workflow. Although designed to target specific protein types, this was not borne out in practice as the peptide markers were effective in detecting proteins present in both the gliadin and glutenin fractions. One of the marker peptides carrying a CD motif (QPFPQPQLPY) performed poorly in the MRM experiments, possibly as a consequence of N-terminal pyroglutamic acid formation, although this is a slow reaction that requires incubation at elevated temperatures, such as 50 °C for at least a week to make an appreciable difference.^46^ However, a second alternative peptide marker carrying a CD motif (RPQQPYPQPQPQY) was retained, together with the peptide GIIQPQQPAQL that contains an IgE epitope. These peptides, together with two others, were synthesized as isotopologues and used as internal standards to develop conversion factors generated from the well-defined Osborne fractions, addressing a gap in existing MS-based gluten detection methods. Various quantitative MS-based gluten detection methods have been developed, but in general, they have not used heavy isotopically labeled peptides as internal standards or applied conversion factors; instead, they have inferred both LODs and LOQs by reference to samples that contained different levels of wheat flour or gluten.^25,27,47^ Of the peptides assessed, the LMW-GS peptide VQQQIPVVQPSIL, using the glutenin conversion factor, gave the most accurate determination of the gluten protein. Peptides RPQQPYPQPQPQY^23–26^ and VQQQIPIVQPSVL^26,27^ have been reported in previous studies, but, unlike this study, they were not used as isotopically labeled standards. Schalk et al.^26^ used a different isotopically labeled α-gliadin peptide, LQLQPFPQPQLPYPQPQP, but did not report the SID series and the associated LODs and LOQ values.

In order to develop a LC-MS/MS method to determine the amount of total gluten proteins in food, it is necessary to establish an effective protein extraction method, ensuring an effective sample preparation that maximizes the generation of marker peptides as well as establishing factors to allow conversion of the results from amount of peptide to amount of protein.^48,49^ The sample preparation protocol applied in this report builds on an evaluation of different extraction methodologies, which have been shown to be highly effective in extracting gluten proteins^17^ and highly effective in releasing these peptide markers as assessed using untargeted mass spectrometry.^40^ Therefore, the current report has focused on assessing two different approaches for deriving conversion factors. The first calculation method used the average molecular weight of the protein fraction from which the marker peptide was derived and the proportion of that fraction in gluten, which has previously been used for MS-based methods for the determination of peanut and cow’s milk protein.^50–52^ However, when applied to the analysis of a set of wheat flour extracts, it grossly underestimated the protein content. For example, whereas α-gliadins have been reported to account for about 33% of total gluten proteins in flour^31^ and would therefore be expected to be present at ∼25 mg/g flour in the gliadin fraction, the estimated amount was 10-fold lower. The second method used the Osborne fractions to derive conversion factors based on the total protein content determined using the Dumas combustion assay. Using this approach, the amounts of gluten protein determined in flour extracts by the MS method matched more closely the levels determined using the Dumas total combustion method. Indeed, the column-level LOQs (fmol on column) indicate that the method has the potential to detect gluten in the range of 10 mg/kg for the best reporter marker, VQQQIPVVQPSIL. The reproducibility of the analysis was similar to that observed when determining the protein using the Dumas method,^17^ confirming that the extraction step contributes significantly to assay variability.

In conclusion, this study has developed a prototype LC–MS/MS method for absolute quantification of wheat gluten, which potentially has sufficient sensitivity to detect and quantify gluten at levels below the 20 mg/kg level, which is currently required for gluten-free claims. The incorporation of isotopically labeled peptide internal standards and the use of conversion factors generated from the well-defined Osborne fraction address the gap in existing MS-based gluten detection methods. Indeed, very few publications have used either reference materials or well-defined protein fractions to generate conversion factors for absolute quantitation of gluten.^26,53^ Future work will focus on validation of the MS method for incurred matrices and the approach to deriving conversion factors, together with ensuring the sample workflow maximizes the release of peptide markers from incurred matrices, as has been done for other food allergens.^48,54,55^ Such validation, and extension to methods employing different sample workflows, is required to determine whether the potential of the MS method described here, and the protocol using protein fractions to provide conversion factors from peptide to protein can be realized in practice.

## Supplementary Material



## Data Availability

Data are available as part of the Supporting Information accompanying this paper. (Xu et al. Supporting Information) which includes S1 Supplementary methods (including Supplementary Tables S1 and S2 and Figures S1 and S2), Supplementary results S1 describing HPLC characterization of protein fractions (including Supplementary Tables S3–S7 and Supplementary Figures S3–S10), and associated Supplementary references. Skyline line files are available on Figshare at 10.48420/28911944.v1.

## Notes

S1 Supplementary methods (Supplementary Tables S1 and S2 and Figures S1 and S2); S1 Supplementary results describing HPLC characterization of protein fractions (Supplementary Tables S3–S7 and Supplementary Figures S3–S10), and associated Supplementary references (PDF)

## Abbreviations

WDEIA: wheat-dependent exercise-induced anaphylaxis
CD: celiac disease
IgE: immunoglobulin E
PWG: prolamin working group
ALGL: albumin and globulin
HMW-GS: high-molecular-weight glutenin subunits
LMW-GS: low-molecular-weight glutenin subunits
AP: alkaline phosphate
DTT: dithiothreitol
ELISA: enzyme-linked immunosorbent assay
FA: formic acid
RP-HPLC: reverse phase-high-performance liquid chromatography
ESI: electrospray ionization
MRM: multiple reaction monitoring
LC-MS/MS: liquid chromatography tandem mass spectrometry
PCA: principal component analysis
ANOVA: analysis of variance
CV: coefficient of variation
MWCO: molecular weight cutoff
PBS: phosphate-buffered saline
SDS-PAGE: sodium dodecyl-sulfate polyacrylamide gel electrophoresis

## References

[1] Mustalahti K., Catassi C., Reunanen A., Fabiani E., Heier M., McMillan S., Murray L., Metzger M. H., Gasparin M., Bravi E., Mäki M. (2010). The prevalence
of celiac disease
in Europe: results of a centralized, international mass screening
project. Ann. Med..

[2] Zuidmeer L., Goldhahn K., Rona R. J., Gislason D., Madsen C., Summers C., Sodergren E., Dahlstrom J., Lindner T., Sigurdardottir S. T. (2008). The prevalence of plant
food allergies: a systematic review. J. Allergy
Clin. Immunol..

[3] Levescot A., Malamut G., Cerf-Bensussan N. (2022). Immunopathogenesis
and environmental
triggers in coeliac disease. Gut.

[4] Feighery C. (1999). Coeliac disease. Br. Med. J..

[5] Scherf K. A., Brockow K., Biedermann T., Koehler P., Wieser H. (2016). Wheat-dependent
exercise-induced anaphylaxis. Clin. Exp. Allergy.

[6] Tundo S., Lupi R., Lafond M., Giardina T., Larre C., Denery-Papini S., Morisset M., Kalunke R., Sestili F., Masci S. (2018). Wheat ATI
CM3, CM16 and 0.28 Allergens Produced in Pichia pastoris Display a Different Eliciting Potential
in Food Allergy to Wheat^‡^. Plants.

[7] Codex . Standard for Foods for Special Dietary Use for Persons Intolerant to Gluten; Codex, 2015.

[8] Rzychon M., Brohee M., Cordeiro F., Haraszi R., Ulberth F., O’Connor G. (2017). The feasibility of harmonizing gluten ELISA measurements. Food Chem..

[9] Colgrave M. L., Byrne K., Blundell M., Howitt C. A. (2016). Identification of
barley-specific peptide markers that persist in processed foods and
are capable of detecting barley contamination by LC-MS/MS. J. Proteomics.

[10] van
den Broeck H. C., Cordewener J. H., Nessen M. A., America A. H., van der Meer I. M. (2015). Label free targeted detection and quantification of
celiac disease immunogenic epitopes by mass spectrometry. J. Chromatogr. A.

[11] Van
Eckert R., Berghofer E., Ciclitira P., Chirdo F., Denery-Papini S., Ellis H., Ferranti P., Goodwin P., Immer U., Mamone G. (2006). Towards
a new gliadin reference material–isolation and characterisation. J. Cereal Sci..

[12] Diaz-Amigo C., Popping B. (2013). Accuracy of ELISA detection methods
for gluten and
reference materials: a realistic assessment. J. Agric. Food Chem..

[13] Lacorn M., Dubois T., Weiss T., Zimmermann L., Schinabeck T. M., Loos-Theisen S., Scherf K. (2022). Determination of Gliadin
as a Measure of Gluten in Food by R5 Sandwich ELISA RIDASCREEN­(R)
Gliadin Matrix Extension: Collaborative Study 2012.01. J. AOAC Int..

[14] Shewry P. R., Parmar S., Miflin B. J. (1983). Extraction,
Separation, and Polymorphism
of the Prolamin Storage Proteins (Secalins) of Rye. Cereal Chem..

[15] Neves M. A. d., Kimura T., Shimizu N., Shiiba K. (2006). Production of alcohol
by simultaneous saccharification and fermentation of low-grade wheat
flour. Braz. Arch. Biol. Technol..

[16] Tatham, A. S. ; Gilbert, S. M. ; Fido, R. J. ; Shewry, P. R. Extraction, Separation, and Purification of Wheat Gluten Proteins and Related Proteins of Barley, Rye, and Oats. Celiac Disease; Springer, 2000; pp 55–73. 10.1385/1-59259-082-9:055 21374432

[17] Daly M., Huang X., Nitride C., Tranquet O., Rogers A., Shewry P. R., Gethings L. A., Mills E. N. C. (2023). A chromatographic
and immunoprofiling approach to optimizing workflows for extraction
of gluten proteins from flour. J. Chromatogr.
B.

[18] Tontisirin, K. ; MacLean, W. C. ; Warwick, P. Food Energy: Methods of Analysis and Conversion Factors, Report of a Technical Workshop, Rome, 3–6 December, 2002; Food and Agriculture Organization of the United Nations, 2003.

[19] Lowry O. H., Rosebrough N. J., Farr A. L., Randall R. J. (1951). Protein measurement
with the Folin phenol reagent. J. Biol. Chem..

[20] Arentz-Hansen H., Körner R., Molberg O., Quarsten H., Vader W., Kooy Y. M. C., Lundin K. E. A., Koning F., Roepstorff P., Sollid L. M., McAdam S. N. (2000). The intestinal T cell response to
α-gliadin in adult celiac disease is focused on a single deamidated
glutamine targeted by tissue transglutaminase. J. Exp. Med..

[21] Qiao S. W., Bergseng E., Molberg O., Jung G., Fleckenstein B., Sollid L. M. (2005). Refining the rules of gliadin T cell epitope binding
to the disease-associated DQ2 molecule in celiac disease: importance
of proline spacing and glutamine deamidation. J. Immunol..

[22] Vader W., Kooy Y., Van Veelen P., De Ru A., Harris D., Benckhuijsen W., Peña S., Mearin L., Drijfhout J. W., Koning F. (2002). The gluten response in children with Celiac disease
is directed toward multiple gliadin and glutenin peptides. Gastroenterology.

[23] Sealey-Voyksner J. A., Khosla C., Voyksner R. D., Jorgenson J. W. (2010). Novel aspects
of quantitation of immunogenic wheat gluten peptides by liquid chromatography-mass
spectrometry/mass spectrometry. J. Chromatogr.
A.

[24] Pompa M., Giuliani M. M., Palermo C., Agriesti F., Centonze D., Flagella Z. (2013). Comparative analysis of gluten proteins
in three durum
wheat cultivars by a proteomic approach. J.
Agric. Food Chem..

[25] Fiedler K. L., McGrath S. C., Callahan J. H., Ross M. M. (2014). Characterization
of grain-specific peptide markers for the detection of gluten by mass
spectrometry. J. Agric. Food Chem..

[26] Schalk K., Koehler P., Scherf K. A. (2018). Targeted
liquid chromatography tandem
mass spectrometry to quantitate wheat gluten using well-defined reference
proteins. PLoS One.

[27] Manfredi A., Mattarozzi M., Giannetto M., Careri M. (2015). Multiplex liquid chromatography-tandem
mass spectrometry for the detection of wheat, oat, barley and rye
prolamins towards the assessment of gluten-free product safety. Anal. Chim. Acta.

[28] Pang Z. Q., Chong J., Zhou G. Y., Morais D. A. D., Chang L., Barrette M., Gauthier C., Jacques P. É., Li S. Z., Xia J. G. (2021). MetaboAnalyst 5.0: narrowing the
gap between raw spectra and functional insights. Nucleic Acids Res..

[29] Miller, J. N. ; Miller, J. C. Statistics and Chemometrics for Analytical Chemistry, 7th ed.; Pearson Education Limited, 2018.

[30] Daly M., Bromilow S. N., Nitride C., Shewry P. R., Gethings L. A., Mills E. N. C. (2020). Mapping Coeliac Toxic Motifs in the
Prolamin Seed Storage
Proteins of Barley, Rye, and Oats Using a Curated Sequence Database. Front. Nutr..

[31] Schalk K., Lexhaller B., Koehler P., Scherf K. A. (2017). Isolation and characterization
of gluten protein types from wheat, rye, barley and oats for use as
reference materials. PLoS One.

[32] Geisslitz S., Shewry P., Brouns F., America A. H. P., Caio G. P. I., Daly M., D’Amico S., De Giorgio R., Gilissen L., Grausgruber H. (2021). Wheat ATIs: Characteristics
and Role in Human Disease. Front. Nutr..

[33] Gilbert S. M., Burnett G. R., Mills E. N. C., Belton P. S., Shewry P. R., Tatham A. S. (2003). Identification of
the wheat seed protein CM3 as a highly
active emulsifier using a novel functional screen. J. Agric. Food Chem..

[34] Shewry P. R., Tatham A. S., Forde J., Kreis M., Miflin B. J. (1986). The Classification
and Nomenclature of Wheat Gluten Proteins - a Reassessment. J. Cereal Sci..

[35] Perez-Gregorio M. R., Dias R., Mateus N., de Freitas V. (2017). Chromatographic
and mass spectrometry analysis of wheat flour prolamins, the causative
compounds of celiac disease. Food Funct..

[36] Wan Y., Gritsch C. S., Hawkesford M. J., Shewry P. R. (2014). Effects of nitrogen
nutrition on the synthesis and deposition of the omega-gliadins of
wheat. Ann. Bot..

[37] Wieser H. (2000). Comparative
investigations of gluten proteins from different wheat species I.
Qualitative and quantitative composition of gluten protein types. Eur. Food Res. Technol..

[38] Morel M. H., Pincemaille J., Chauveau E., Louhichi A., Violleau F., Menut P., Ramos L., Banc A. (2020). Insight into gluten
structure in a mild chaotropic solvent by asymmetrical flow field-flow
fractionation (AsFlFFF) and evidence of non-covalent assemblies between
glutenin and ω-gliadin. Food Hydrocolloids.

[39] Huet A. C., Paulus M., Henrottin J., Brossard C., Tranquet O., Bernard H., Pilolli R., Nitride C., Larre C., Adel-Patient K. (2022). Development of incurred chocolate bars and
broth powder with six fully characterised food allergens as test materials
for food allergen analysis. Anal. Bioanal. Chem..

[40] Daly M. E., Huang X., Nitride C., Hughes C., Tanskanen J., Shewry P. R., Gethings L. A., Mills E. N. C. (2025). Proteomic Profiling
of Celiac-Toxic Motifs and Allergens in Cereals Containing Gluten. J. Proteome Res..

[41] Buckee G. K. (1994). Determination
of total nitrogen in barley, malt and beer by Kjeldahl procedures
and the dumas combustion methodcollaborative trial. J. Inst. Brew..

[42] Miller E. L., Bimbo A. P., Barlow S. M., Sheridan B. (2007). Repeatability
and reproducibility of determination of the nitrogen content of fishmeal
by the combustion (Dumas) method and comparison with the Kjeldahl
method: interlaboratory study. J. AOAC Int..

[43] DuPont F. M., Chan R., Lopez R., Vensel W. H. (2005). Sequential extraction
and quantitative recovery of gliadins, glutenins, and other proteins
from small samples of wheat flour. J. Agric.
Food Chem..

[44] Belton P. (1999). Mini review:
on the elasticity of wheat gluten. J. Cereal
Sci..

[45] Sander I., Rozynek P., Rihs H. P., van Kampen V., Chew F. T., Lee W. S., Kotschy-Lang N., Merget R., Bruning T., Raulf-Heimsoth M. (2011). Multiple wheat
flour allergens and cross-reactive carbohydrate determinants bind
IgE in baker’s asthma. Allergy.

[46] Bersin L. M., Patel S. M., Topp E. M. (2021). Effect of ’pH’ on the
Rate of Pyroglutamate Formation in Solution and Lyophilized Solids. Mol. Pharmaceutics.

[47] Colgrave M. L., Goswami H., Byrne K., Blundell M., Howitt C. A., Tanner G. J. (2015). Proteomic profiling
of 16 cereal grains and the application
of targeted proteomics to detect wheat contamination. J. Proteome Res..

[48] Martinez-Esteso M. J., O’Connor G., Norgaard J., Breidbach A., Brohee M., Cubero-Leon E., Nitride C., Robouch P., Emons H. (2020). A reference method
for determining the total allergenic protein content
in a processed food: the case of milk in cookies as proof of concept. Anal. Bioanal. Chem..

[49] Nitride C., Norgaard J., Omar J., Emons H., Esteso M. M., O’Connor G. (2019). An assessment
of the impact of extraction and digestion
protocols on multiplexed targeted protein quantification by mass spectrometry
for egg and milk allergens. Anal. Bioanal. Chem..

[50] Martínez-Esteso M. J., Norgaard J., Brohee M., Haraszi R., Maquet A., O’Connor G. (2016). Defining the wheat gluten peptide fingerprint via a
discovery and targeted proteomics approach. J. Proteomics.

[51] Sayers R. L., Gethings L. A., Lee V., Balasundaram A., Johnson P. E., Marsh J. A., Wallace A., Brown H., Rogers A., Langridge J. I., Mills E. N. C. (2018). Microfluidic
Separation Coupled to Mass Spectrometry for Quantification of Peanut
Allergens in a Complex Food Matrix. J. Proteome
Res..

[52] Parker C. H., Khuda S. E., Pereira M., Ross M. M., Fu T. J., Fan X., Wu Y., Williams K. M., DeVries J., Pulvermacher B. (2015). Multi-allergen Quantitation
and the Impact of Thermal Treatment in
Industry-Processed Baked Goods by ELISA and Liquid Chromatography-Tandem
Mass Spectrometry. J. Agric. Food Chem..

[53] Schalk K., Koehler P., Scherf K. A. (2018). Quantitation of Specific Barley,
Rye, and Oat Marker Peptides by Targeted Liquid Chromatography-Mass
Spectrometry To Determine Gluten Concentrations. J. Agric. Food Chem..

[54] Henrottin J., Pilolli R., Huet A.-C., van Poucke C., Nitride C., De Loose M., Tranquet O., Larré C., Adel-Patient K., Bernard H. (2023). Optimization
of a sample
preparation workflow based on UHPLC-MS/MS method for multi-allergen
detection in chocolate: An outcome of the ThRAll project. Food Control.

[55] Pilolli R., Lamonaca A., Nitride C., De Angelis E., van Poucke C., Gillard N., Huet A. C., De Loose M., Henrottin J., Mills E. C. N., Monaci L. (2024). In-house validation
of an LC-MS method for the multiplexed quantitative determination
of total allergenic food in chocolate. Anal.
Bioanal. Chem..

