# Supplementary material for: From Peptide to Protein: Development of Conversion Factors for the Quantification of Gluten Using Targeted Mass Spectrometry
Source: J Agric Food Chem. 2025 May 22;73(22):14099–111. doi: 10.1021/acs.jafc.4c12344 (PMC12147133; doi:10.1021/acs.jafc.4c12344)
Supplement: Supplementary file 1 [file jf4c12344_si_001.pdf]

Supplementary Information

From peptide to protein: Development of conversion factors for quantification of gluten using targeted mass spectrometry.

Qianying Xu[1], Matthew Daly[2], Chiara Nitride[3], Olivier Tranquet[4], Adrian Rogers[5], Joan Bartra-Tomas[6], Angela Simpson[7], Peter R. Shewry[8], Lee A Gethings[1,2], EN Clare Mills[1, 9*]

[1] Division of Immunology, Immunity to Infection and Respiratory Medicine, School of Biological Sciences, Manchester Institute of Biotechnology, University of Manchester, Manchester, M1 7DN, United Kingdom

[2] Waters Corporation, Wilmslow, SK9 4AX, United Kingdom

[3] Department of Agricultural Sciences, University of Naples Federico II, Portici, Italy

[4] INRAE, Aix Marseille University, UMR1163 Biodiversit´e Et Biotechnologie Fongiques, (BBF), UMR1163, 13009 Marseille, France

[5] Bio-Check, St. Asaph Denbighshire, LL17 0JA, United Kingdom

[6] Allergy Department. Hospital Clinic, University of Barcelona, 08036 Barcelona, Spain; Clinical and Experimental Respiratory Immunoallergy, Institut Investigacions Biomediques August Pi I Sunyer (IDIBAPS), 08036 Barcelona, Spain.

[7] Division of Immunology, Immunity to Infection and Respiratory Medicine, School of Biological Sciences, Manchester University NHS Foundation Trust, University of Manchester, Manchester, M23 9LT, United Kingdom

[8] Rothamsted Research, Harpenden, AL5 2JQ, United Kingdom

[9] School of Biosciences, University of Surrey, Guildford, GU2 7XH, United Kingdom

* Corresponding author. E-mail address: clare.mills@manchester.ac.uk; clare.mills@surrey.ac.uk

Table of contents

S1 SUPPLEMENTARY METHODS...3

TABLE. S1. THE INFORMATION FOR ANIMAL ANTIBODIES USED IN IMMUNOBLOT. 3

TABLE. S2. CLINICAL INFORMATION FROM A WHEAT-ALLERGIC PATIENT PANEL (A-W). PATIENTS WERE FROM SPANISH OR UK* POPULATIONS. 4

S1. REVERSED PHASE-HIGH PERFORMANCE LIQUID CHROMATOGRAPHY (RP-HPLC) 6

FIGURE. S1. THE SEQUENTIAL EXTRACTION FLOWSHEET FOR THE WHEAT PROTEIN FRACTIONATION. *THE RATIO BETWEEN SAMPLE AND BUFFER REFERS TO THE STARTING WEIGHT OF DEFATTED FLOUR 7

FIGURE. S2. THE WORKFLOW SHEET FOR MASS SPECTROMETRY SAMPLE PREPARATION (THE REDUCTION, ALKYLATION AND DIGESTION)...8

SUPPLEMENTARY RESULTS...8

TABLE. S3. THE MEAN, STANDARD DEVIATION AND COEFFICIENT OF VARIATION OF PEAK AREA FOR ALGL, GLIADIN AND GLUTENIN FRACTIONS AGAINST EACH PEPTIDE 9

TABLE. S4. THE TRANSITION ION RATIO FOR THE SIDS. QUANTIFIER IONS ARE IN BOLD; PEAK HEIGHT INTENSITIES BELOW 3×S/N ARE SHADED IN GREY. 10

TABLE. S5. STANDARD CURVE LINEAR FIT, THE LIMIT OF DETECTION AND LIMIT OF QUANTITATION...11

TABLE. S6. THE TRANSITION ION RATIO FOR THE OSBORNE FRACTIONS AND ONE/TWO-STEP EXTRACTED SAMPLES. QUANTIFIER IONS ARE IN BOLD. 13

TABLE S7. THE CONVERSION FACTOR FOR ALGL FRACTION. 14

FIGURE. S3. THE LC-MS CHROMATOGRAM OBTAINED FROM THREE FRACTIONS OF PEPTIDE1-9 (A-I). 24

FIGURE. S4. THE NORMALISED BINDING INTENSITY IDENTIFIED FROM IGE IMMUNOBLOTS OF GLIADIN AND GLUTENIN AT MR ~ 70, 55, AND 40 KDA 25

FIGURE. S5. THE IGE-REACTIVITY OF THE OSBORNE FRACTIONS AGAINST PATIENTS WITH IGE-MEDIATED WHEAT ALLERGY (SERA A-W)..26

SUPPLEMENTARY RESULTS S1 HPLC CHARACTERISATION OF PROTEIN FRACTIONS.........31

FIGURE. S7. RP-HPLC ANALYSIS OF THE ALGL FRACTION. 32

FIGURE. S8. RP-HPLC ANALYSIS OF THE GLIADIN FRACTION..33

FIGURE. S9. RP-HPLC ANALYSIS OF THE GLUTENIN FRACTION. 33

FIGURE. S10. THE CHROMATOGRAM OF PWG-GLIADIN. 34

SUPPLEMENTARY REFERENCES...35

S1 Supplementary methods

Table. S1. The information for animal antibodies used in immunoblot.

Antibody	Target proteins	Binding epitopes	Supplier/reference
Mouse monoclonal anti-gluten, R5	gluten protein	QQQFP, QQPFP, LQPFP, QLPFP	Operon (Spain) [1]
Mouse monoclonal anti-33mer, G12	33-mer	QPQLPY	Gift of Adrian Rogers [2]
Mouse monoclonal anti-gluten, IFRN 0065	Gliadins<br>LWM-GS	QQSF	[3]
Mouse monoclonal anti-gltuen, IFRN 0610	Gliadins<br>LWM-GS	QPFP	[3]
Mouse monoclonal anit-gluten, IFRN 1602	HMW-GS		[4]
Mouse monoclonal anti- ω5-gliadins, ONT18A5	ω5-gliadins	ω5-gliadin N terminus	Gift of Oliver Tranquet
Rabbit polyclonal anit-CM3	α-amylase inhibitor CM3	KLPEWMTSASIYSPGK	Gift of Peter Shewry
Goat polyclonal anti-mouse, AP conjugation	Mouse IgG	-	ThermoFisher Scientific (UK)
Goat polyclonal anti-rabbit, AP conjugation	Rabbit IgG	-	ThermoFisher Scientific (UK)
Mouse monoclonal anti-human IgE, AP conjugation	Human IgE Fc region	-	Abcam (UK)

Table. S2. Clinical information from a wheat-allergic patient panel (A-W). Patients were from Spanish or UK* populations.

Patient	Gender	Age (yrs)	Clinical Data	Co-factors	Total IgE (kU/L)	Wheat Specific IgE (kU/L)	ω-5 gliadin IgE (kU/L)	Wheat SPT (mm)	Gliadin/wheat flour* SPT (mm)
A	M	69	Anaphylaxis (shock)	Exercise, NSAID	495	0.56	6.46	4 x 4	8 x 8
B	M	56	Anaphylaxis	Exercise, NSAID	663	**	13.7	0 x 0	6 x 6
C	M	57	Anaphylaxis	Exercise, NSAID	263	0.71	30.8	7	8 x 8
D	F	35	Acute urticarial	Exercise	87	**	4.99	**	3 x 3
E	F	66	Anaphylaxis (shock)	Exercise	347	2.34	0.4	0 x 0	0 x 0
F	M	35	Acute urticarial	NSAID	99.4	**	0.75	**	0 x 0
G	F	39	Anaphylaxis	Exercise, NSAID	69	**	5.11	**	5 x 5
H	F	28	Anaphylaxis	Exercise	168	1.57	0.01	0 x 0	7 x 7
I	M	64	Anaphylaxis	Exercise, NSAID	269	**	0.45	0 x 0	0 x 0
J	M	60	Anaphylaxis	Exercise	247	0.86	17.5	0 x 0	6 x 5
K	M	39	Anaphylaxis	Exercise, NSAID	162	**	7.16	**	3 x 3

L	M	50	Anaphylaxis	NSAID	164	**	0.96	**	0 x 0
M	M	34	Anaphylaxis	Exercise, NSAID	974	**	8.96	3 x 3	3 x 3
N	F	45	Angioedema	NSAID	**	**	5.44	3	8 x 8
O	F	55	Acute urticarial / Anaphylaxis	Exercise	42.4	0.6	3.99	**	5 x 5
P	M	44	Acute urticarial	Exercise	186	**	7.56	0 x 0	7 x 7
Q	M	18	Anaphylaxis (shock)	None	346	> 100	2.23	8 x 8	8 x 8
R	M	42	Angioedema	NSAID	103	**	0.5	0 x 0	3 x 3
S*	F	42	Acute urticarial / Anaphylaxis	**	**	< 0.3	14	5	3 x 3
T*	F	46	Acute urticarial / Anaphylaxis	Exercise	**	**	9.6	**	**
U*	F	44	Acute urticarial / Angioedema / Anaphylaxis	**	2400	7.6	47	**	**
V*	F	39	Acute urticarial / Angioedema / Anaphylaxis	**	**	**	1.4	**	**

W*	F	59	Acute urticarial	**	250	< 0.4	4	0	4 x 4

Note: Symptoms included anaphylaxis, acute urticarial and angioedema after consumption of wheat-containing foods and co-factors of exercise or non-steroidal anti-inflammatory drugs (NSAIDs). M-males; F-female; SPT-skin prick test, performed using wheat extract (all patients), gliadin (Spanish population) or wheat flour (UK population). ** Data currently unavailable.

S1. Reversed Phase-High Performance Liquid Chromatography (RP-HPLC)

Wheat flour fractions were dissolved at 1 mg/mL in either 0.5M NaCl (ALGL),70% (v/v) aqueous ethanol (gliadin fraction) or 50% (v/v) aqueous propan-2-ol, 60 mM DTT and 1% (v/v) acetic acid (glutenin fraction) and filtered through 0.45 µm syringe filter prior to analysis. RP-HPLC was performed using a Shimadzu Prominence HPLC system (Shimadzu, Milton Keynes, UK) equipped with a Phenomenex 5 µm Jupiter® C18 300Å 4.6 mm ×250 mm (Phenomenex, Macclesfield, UK). The column temperature was set at 40 °C, and 100 µL of sample was injected. Separations were performed using a flowrate of 1.2 mL/min and eluted proteins and peptides detected by their absorbance at 210 nm. The elution gradient employed HPLC grade water with 0.1% (v/v) formic acid (FA, mobile phase A) and acetonitrile containing 0.1% (v/v) FA (mobile phase B). The gradient was as follows: 0 min 24% mobile phase B, 20 min 56% mobile phase B, 24-26min 90% mobile phase B, 32-36min 24% mobile phase B [5]. Peak fractions were collected and pooled. Data were exported to .txt files and the chromatograms were visualised through GraphPad Prism (version 9.1.2).

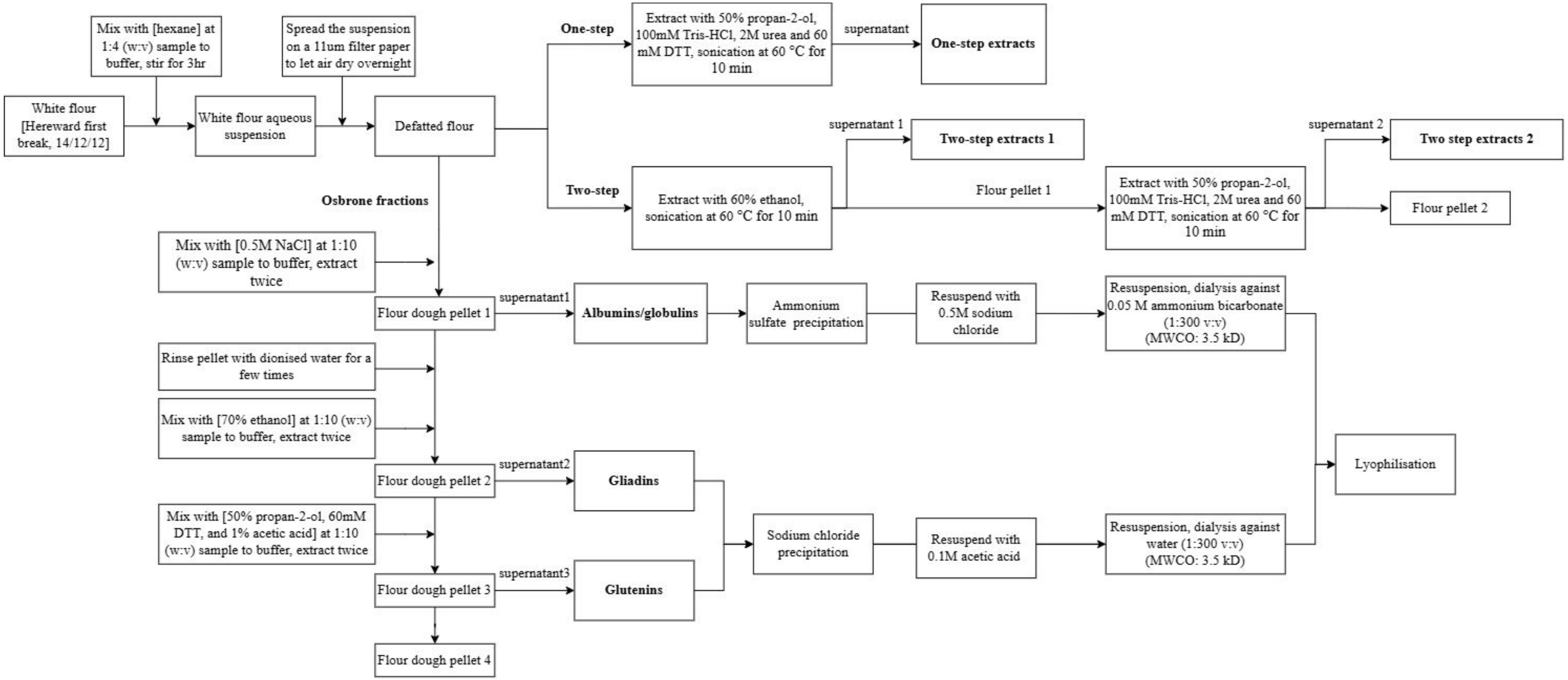

Figure. S1. The sequential extraction flowsheet for the wheat protein fractionation. *The ratio between sample and buffer refers to the starting weight of defatted flour

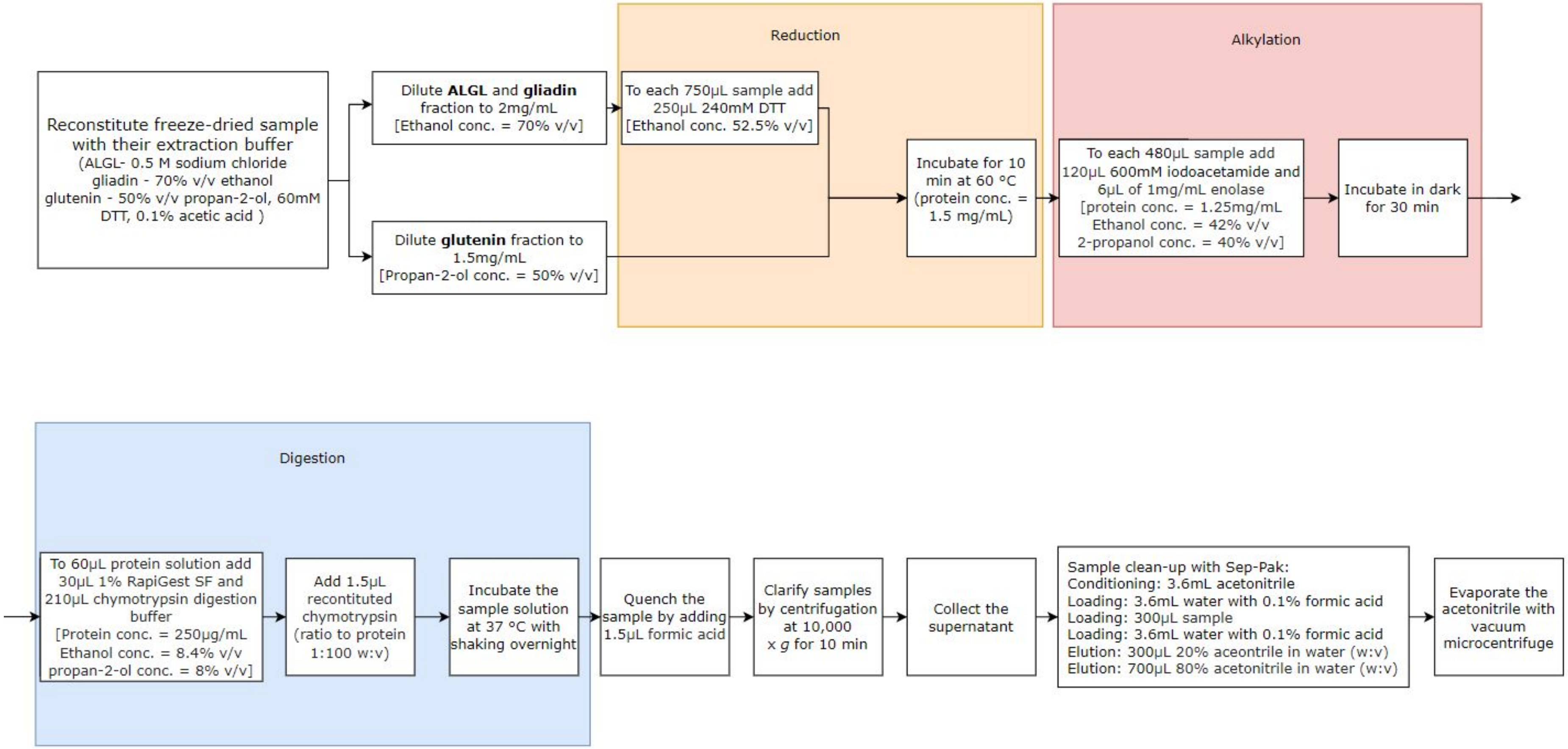

Figure. S2. The workflow sheet for mass spectrometry sample preparation (the reduction, alkylation and digestion).

Supplementary results

Table. S3. The mean, standard deviation and coefficient of variation of peak area for ALGL, gliadin and glutenin fractions against each peptide

Peptides	ALGL (peak area)			Gliadin (peak area)			Glutenin (peak area)		
	Mean	SD	CV %	Mean	SD	CV %	Mean	SD	CV %
P1: RPQQPYPQPQPQY	6.38E+06	3.72E+05	5.83	4.93E+07	1.64E+06	3.33	6.84E+06	3.70E+05	5.41
P2: QPFPQPQLPY	7.26E+05	6.63E+04	9.13	-	-	-	-	-	-
P3: IPPHCSTTIAPF	5.92E+05	1.29E+05	21.77	5.97E+06	4.79E+05	8.03	5.37E+06	2.32E+05	4.32
P4: ASIVAGISGQ	5.22E+04	7.07E+03	13.55	1.08E+07	9.22E+05	8.54	1.55E+06	1.87E+05	12.02
P5: ASIVAGIGGQ	3.94E+05	5.81E+04	14.75	6.16E+07	1.72E+06	2.79	1.32E+07	7.87E+05	5.95
P6: GIIQPQQPAQL	1.50E+07	1.09E+06	7.25	2.41E+07	2.73E+06	11.31	5.92E+06	5.48E+05	9.25
P7: VQQQIPVVQPSIL	7.22E+06	5.06E+05	7.01	1.65E+08	1.12E+07	6.76	7.65E+07	5.38E+06	7.03
P8: GVGTGVGAY	-	-	-	4.00E+06	2.80E+05	7.01	2.35E+06	1.41E+05	5.99
P9: GQCVSQPQQQSQQQL	5.35E+06	5.12E+05	9.59	4.60E+07	2.35E+06	5.11	1.21E+07	5.35E+05	4.41

Table. S4. The transition ion ratio for the SIDs. *Quantifier ions are in **bold**; peak height intensities below 3×S/N are shaded in grey.*

Peptide marker	Transition ion	Transition ion ratio (Mean ± SD, n=3)							
		2 fmol	5 fmol	10 fmol	25 fmol	50 fmol	100 fmol	250 fmol	500 fmol
P1: RPQQPYPQPQPQY	**[y3] - 407.1925**	**59.8% ± 0.9**	**59.8% ± 0.6**	**60.7% ± 2.0**	**58.7% ± 0.1**	**57.8% ± 0.3**	**59.3 % ± 0.7**	**58.2% ± 0.6**	**58.5 % ± 0.3**
	[y5] - 632.3039	9.1% ± 0.4	9.3% ± 0.9	8.5% 0.1	9.8% ± 0.2	9.8% ± 0.3	9.7% ± 0.3	9.8% ± 0.0	10.1% ± 0.2
	[b8] - 995.5057	12.9% ± 0.8	13.5% ± 0.2	12.5% ± 0.4	12.8% ± 0.3	13.5% ± 0.4	12.8% ± 0.1	13.0% ± 0.3	13.3% ± 0.1
	[b10] - 1220.6171	18.2% ± 1.4	17.4% ± 0.3	18.3% ± 1.8	18.7% ± 0.3	18.9% ± 0.1	18.2% ± 0.4	18.9 % ± 0.3	18.0% ± 0.2
P2: QPFPQPQLPY	[b5] - 598.298	10.3% ± 2.5	9.0% ± 1.5	9.9% ± 0.4	12.8% ± 0.4	12.8% ± 0.2	12.2% ± 0.3	12.9% ± 0.2	13.4% ± 0.1
	**[b8] - 936.4938**	**85.8% ± 3.3**	**82.8% ± 0.9**	**82.3% ± 0.8**	**78.5% ± 1.2**	**78.5% ± 0.4**	**78.7% ± 0.5**	**77.8% ± 0.1**	**77.3% ± 0.1**
	[b7] - 823.409	3.9% ± 2.2	8.3% ± 2.3	7.8% ± 0.4	8.7% ± 0.8	8.7% ± 0.2	9.1% ± 0.2	9.3% ± 0.3	9.3% ± 0.2
P6: GIIQPQQPAQL	**[y7] - 781.4203**	**43.7% ± 1.6**	**42.7% ± 0.9**	**43.6% ± 1.0**	**42.9% ± 1.1**	**43.3% ± 0.7**	**42.8% ± 0.6**	**43.0 % ± 0.2**	**43.1% ± 0.4**
	[b7] - 765.4254	17.1 % ± 0.9	16.6% ± 0.8	15.9% ± 0.2	16.6% ± 0.5	16.5% ± 0.1	16.8% ± 0.2	16.5% ± 0.0	16.3% ± 0.1
	[b4] - 428.2504	39.2% ± 1.4	40.7% ± 0.4	40.4% ± 0.9	40.5% ± 0.8	40.1% ± 0.1	42.8% ± 0.6	43.0% ± 0.2	43.1% ± 0.4

P7: VQQQIPVVQPSIL	**[b5] - 597.3355**	**33.6% ± 3.9**	**36.3% ± 1.6**	**35.3% ± 1.0**	**35.1% ± 1.0**	**35.3% ± 0.9**	**34.8% ± 0.5**	**34.3% ± 0.7**	**35.0% ± 0.7**
	[y4] - 429.2708	25.5% ± 1.2	21.2% ± 2.6	23.4% ± 0.5	24.6% ± 1.2	21.4% ± 0.4	22.7% ± 1.3	22.3% ± 0.8	22.1% ± 0.4
	[y8] - 852.5189	27.7% ± 3.5	29.2% ± 1.0	28.0% ±1.3	26.6% ± 0.8	29.3% ± 0.8	26.9% ± 0.7	27.2% ± 0.3	26.4% ± 0.4
	[y5] - 557.3293	13.2% ± 1.3	13.4% ± 1.6	13.3% ± 0.9	13.7% ± 0.4	13.9% ± 0.4	15.5% ± 0.3	16.2% ± 0.2	16.5% ± 0.0

Table. S5. Standard curve linear fit, the limit of detection and limit of quantitation

Peptide marker	Regression line	R^2	Limit of Detection fmol peptide on column	Limit of Quantitation fmol peptide on column
Buffer (5% v/v acetonitrile)				
P1: RPQQPYPQPQPQY	Slope: 0.065, intercept: -0.052	0.99	3.1	9.4
P2: QPFPQPQLPY	Slope: 0.041, intercept: -0.17	0.99	5.9	17.6
P6: GIIQPQQPAQL	Slope: 0.045, intercept: 0.081	0.99	1.9	5.8

P7: VQQQIPVVQPSIL	Slope: 0.037, intercept: -0.16	0.99	3.1	9.3
		Gluten-free matrix		
P1: RPQQPYPQPQPQY	Slope: 0.067, intercept: 0.11	0.99	4.4	13.2
P2: QPFPQPQLPY	Slope: 0.046, intercept: -0.16	0.99	6.9	20.7
P6: GIIQPQQPAQL	Slope: 0.047, intercept: 0.046	0.99	2.1	6.2
P7: VQQQIPVVQPSIL	Slope: 0.033, intercept: -0.04	0.99	2.6	7.9

Table. S6. The transition ion ratio for the Osborne fractions and one/two-step extracted samples. Quantifier ions are in **bold**.

Peptide marker	Transition ion	ALGL	Gliadin	Glutenin	One step	Two step 1	Two step 2
P1: RPQQPYPQPQPQY	**[y3] - 407.1925**	**59.5% ± 0.4**	**61.0% ± 0.6**	**60.3% ± 0.8**	**61.0% ± 0.5**	**61.8% ± 0.7**	**61.2% ± 0.8**
	[y5] - 632.3039	9.8% ± 0.2	9.7% ± 0.2	9.7% ± 0.3	9.7% ± 0.2	9.3% ± 0.2	9.9% ± 0.2
	[b8] - 995.5057	12.9% ± 0.2	12.3% ± 0.2	12.5% ± 0.2	12.3% ± 0.2	12.1% ± 0.3	12.2% ± 0.2
	[b10] - 1220.6171	17.8% ± 0.3	17.0% ± 0.3	17.5% ± 0.5	17.0% ± 0.2	16.8% ± 0.4	16.6% ± 0.5
P2: QPFPQPQLPY	[b5] - 598.298	17.8% ± 2.0	45.7% ± 11.2	98.2% ± 1.1	96.7% ± 2.4	79.7% ± 4.0	48.9% ± 11.9
	**[b8] - 936.4938**	**74.9% ± 2.3**	**53.2% ± 10.5**	**1.6% ± 1.3**	**3.0% ± 2.3**	**19.4% ± 3.9**	**48.8% ± 11.1**
	[b7] - 823.409	7.3% ± 0.4	1.1% ± 1.0	0.2% ± 0.4	0.3% ± 0.5	0.9% ± 0.9	2.3% ± 2.6
P6: GIIQPQQPAQL	**[y7] - 781.4203**	**42.8% ± 0.4**	**42.3% ± 0.2**	**42.0% ± 0.4**	**42.6% ± 0.9**	**42.1% ± 0.4**	**42.2% ± 0.4**
	[b7] - 765.4254	16.2% ± 0.2	15.6% ± 0.3	15.6% ± 0.2	15.7% ± 0.4	15.1% ± 0.3	15.4% ± 0.4
	[b4] - 428.2504	41.0% ± 0.3	42.1% ± 0.3	42.3% ± 0.4	41.7% ± 1.1	428. % ± 0.6	42.4% ± 0.4

P7: VQQQIPVVQPSIL	**[b5] - 597.3355**	**36.5% ± 0.4**	**34.1% ± 0.4**	**35.6% ± 0.4**	**35.7% ± 0.3**	**34.4% ± 0.4**	**35.0% ± 0.7**
	[y4] - 429.2708	21.1% ± 0.9	24.9% ± 0.3	21.9% ± 0.5	22.0% ± 0.4	24.9% ± 0.3	24.5% ± 1.2
	[y8] - 852.5189	27.2% ± 0.6	24.3% ± 0.2	25.2% ± 0.5	25.1% ± 0.2	24.4% ± 0.3	24.3% ± 0.5
	[y5] - 557.3293	15.1% ± 1.0	16.7% ± 0.3	17.3% ± 0.2	17.2% ± 0.1	16.3% ± 0.2	16.1% ± 0.4

Table S7. The conversion factor for ALGL fraction.

Peptide marker	Protein type	ALGL fraction (ng gluten protein/fmol peptide)
P1: RPQQPYPQPQPQY	α-gliadin	10.57
P6: GIIQPQQPAQL	g-gliadin	5.40
P7: VQQQIPVVQPSIL	LMW-GS	9.41

a

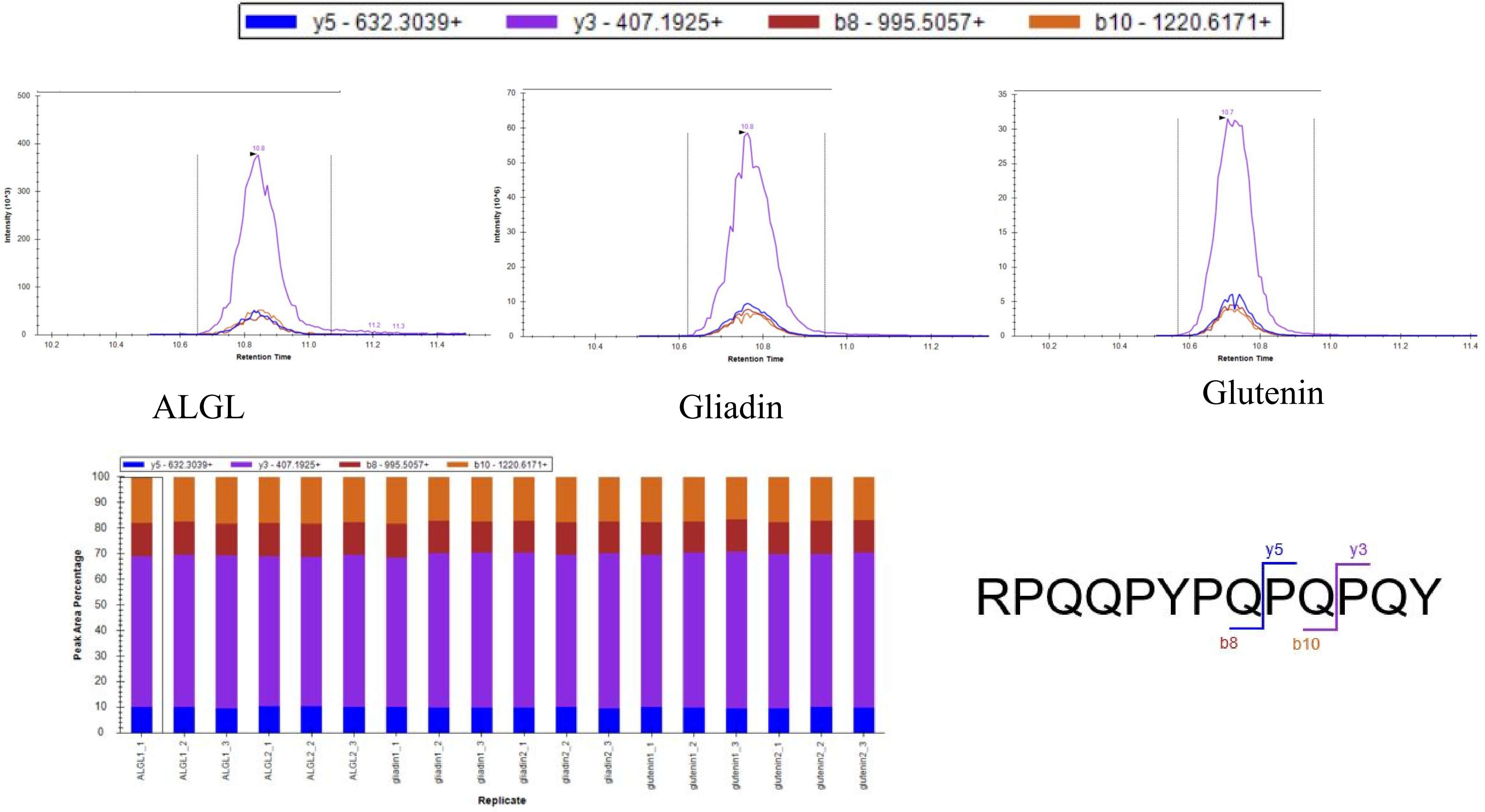

y5 - 632.3039+
y3 - 407.1925+
b8 - 995.5057+
b10 - 1220.6171+
Intensity (10^3)
Intensity (10^6)
Retention Time
ALGL
Gliadin
Glutenin
Peak Area Percentage
Replicate
ALGL1_1
ALGL1_2
ALGL1_3
ALGL2_1
ALGL2_2
ALGL2_3
gliadin1_1
gliadin1_2
gliadin1_3
gliadin2_1
gliadin2_2
gliadin2_3
glutenin1_1
glutenin1_2
glutenin1_3
glutenin2_1
glutenin2_2
glutenin2_3
RPQQPYPQPQPQY
y5
y3
b8
b10

b

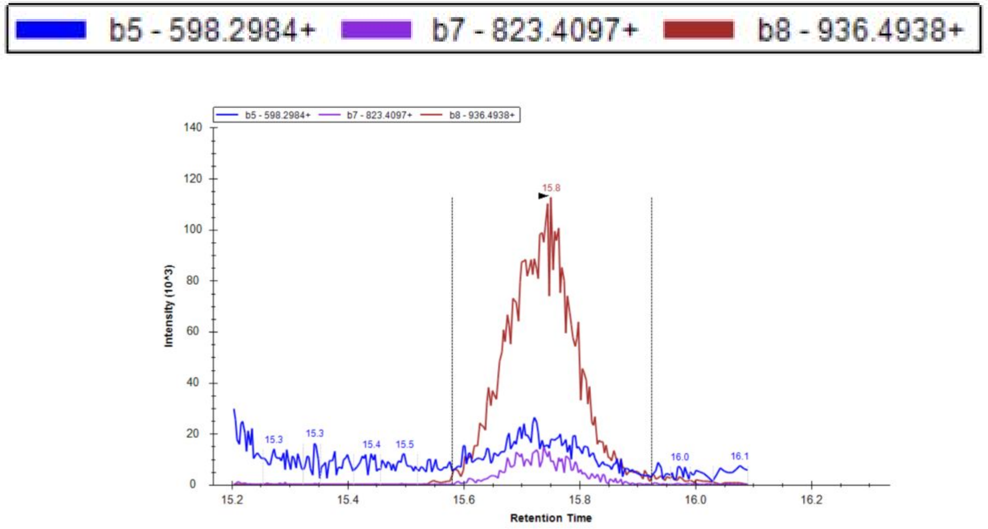
b5 - 598.2984+
b7 - 823.4097+
b8 - 936.4938+
Intensity (10^3)
Retention Time
15.8

ALGL

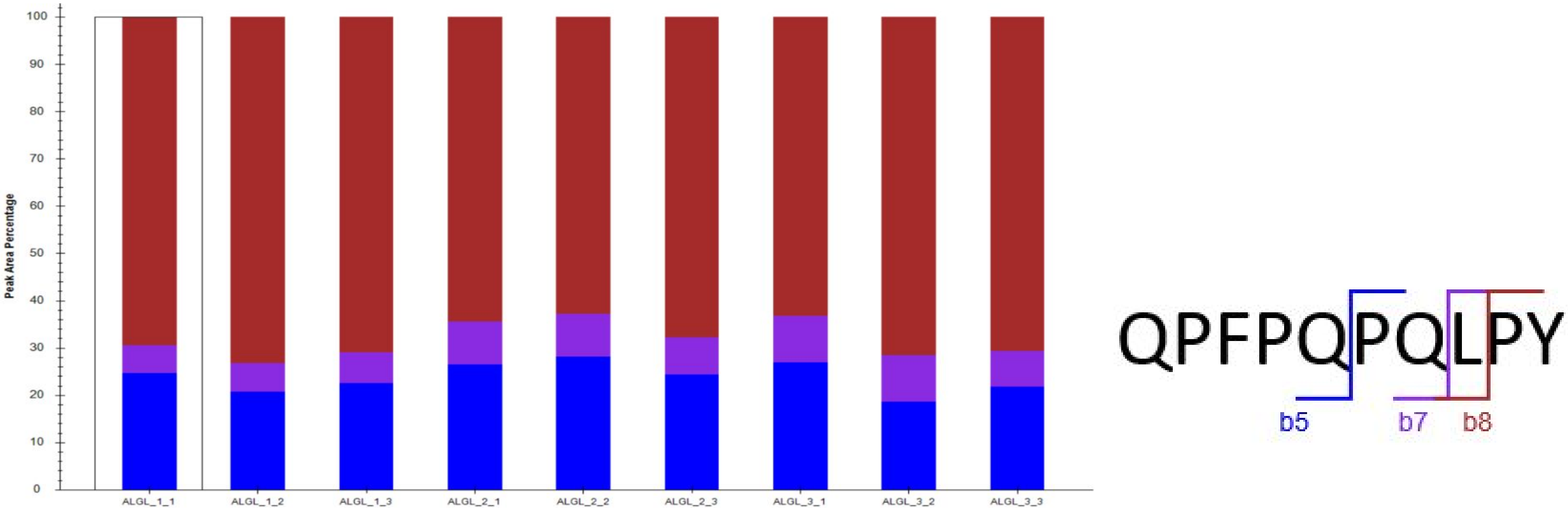
Peak Area Percentage
ALGL_1_1
ALGL_1_2
ALGL_1_3
ALGL_2_1
ALGL_2_2
ALGL_2_3
ALGL_3_1
ALGL_3_2
ALGL_3_3
QPFPQPQLPY
b5
b7
b8

c

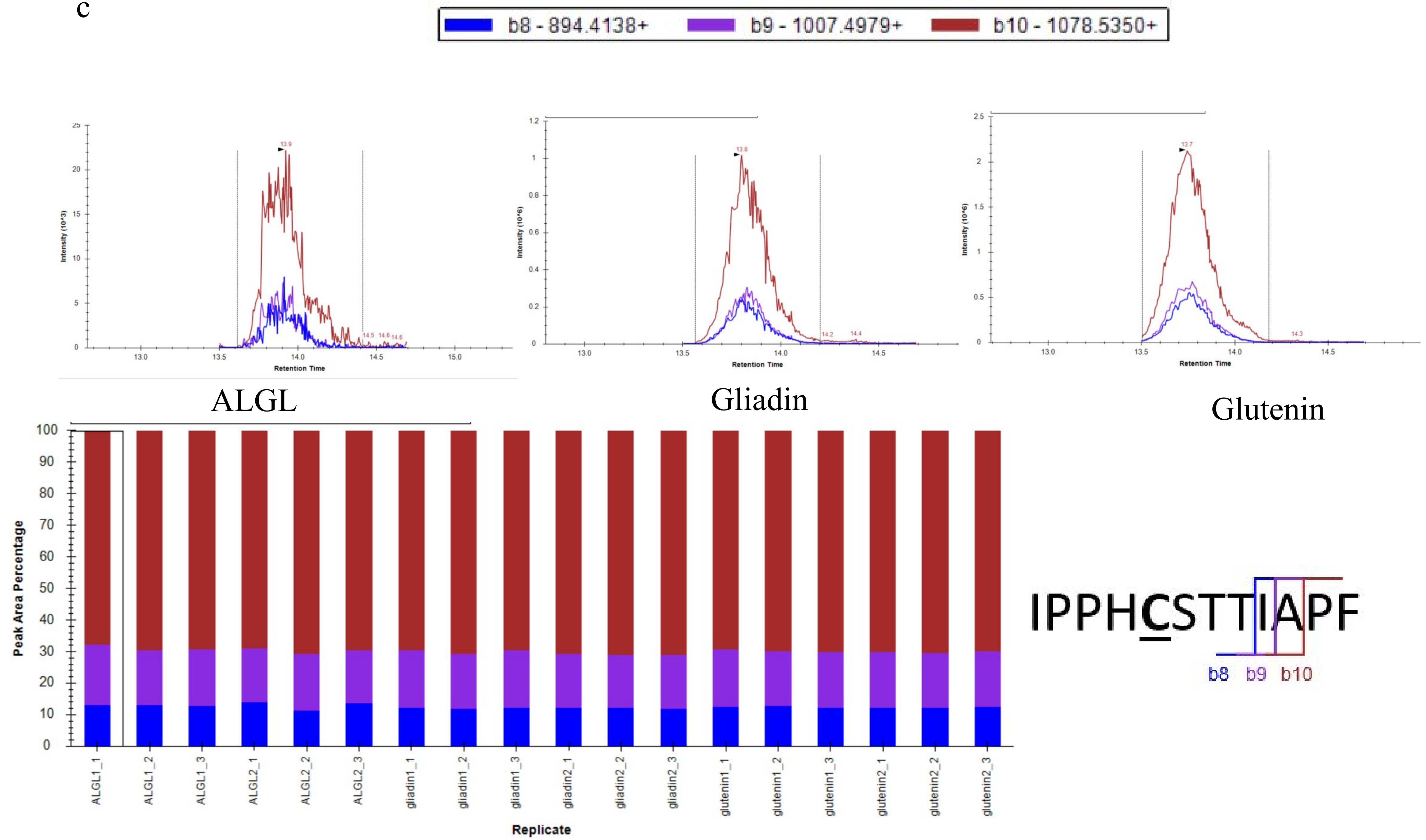

b8 - 894.4138+
b9 - 1007.4979+
b10 - 1078.5350+
Intensity (10^3)
Intensity (10^6)
Retention Time
ALGL
Gliadin
Glutenin
Peak Area Percentage
Replicate
ALGL1_1
ALGL1_2
ALGL1_3
ALGL2_1
ALGL2_2
ALGL2_3
gliadin1_1
gliadin1_2
gliadin1_3
gliadin2_1
gliadin2_2
gliadin2_3
glutenin1_1
glutenin1_2
glutenin1_3
glutenin2_1
glutenin2_2
glutenin2_3
IPPHCSTTIAPF
b8
b9
b10

d

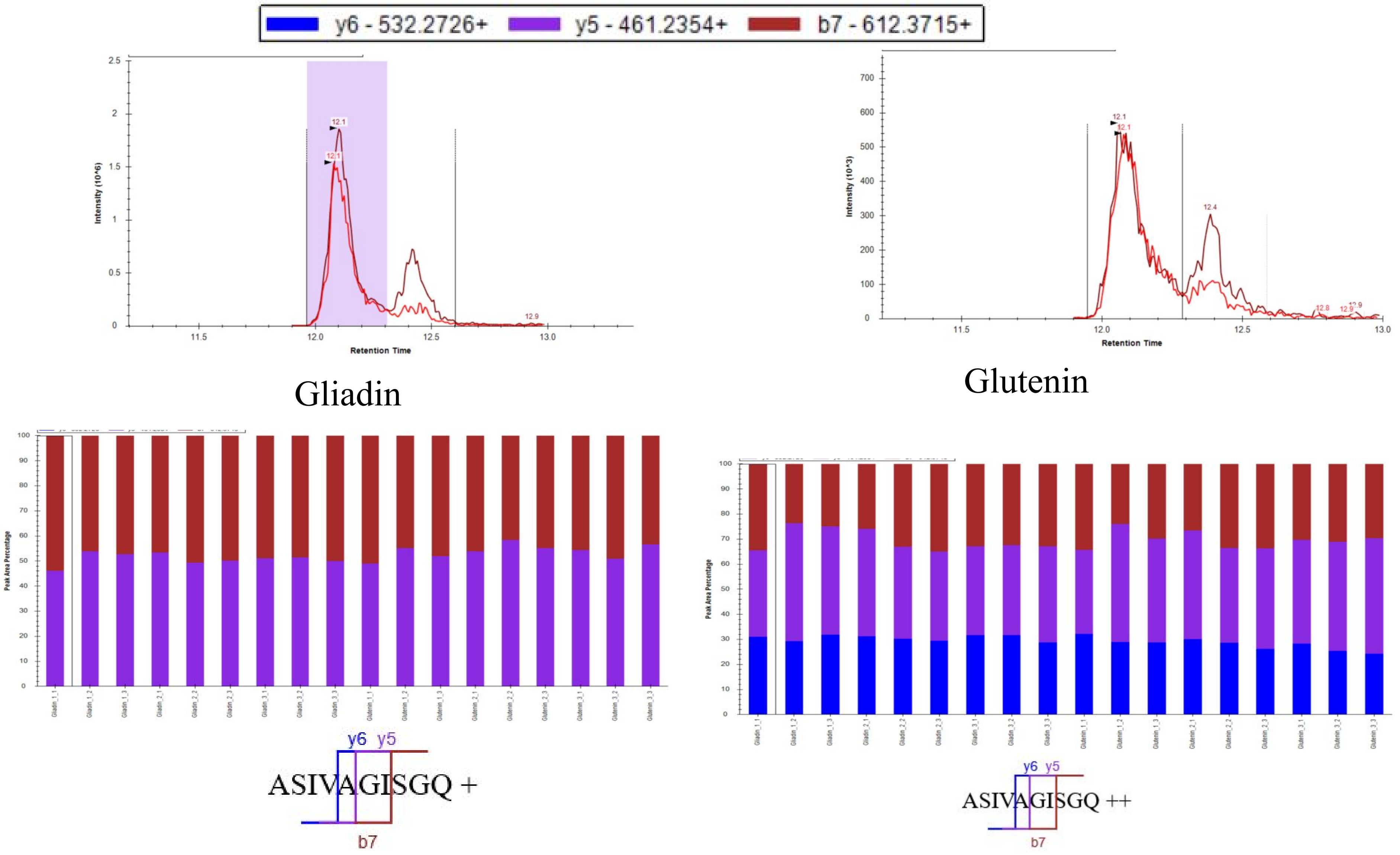

y6 - 532.2726+
y5 - 461.2354+
b7 - 612.3715+
Intensity (10^6)
Retention Time
Intensity (10^3)
Retention Time
Gliadin
Glutenin
Peak Area Percentage
Peak Area Percentage
y6 y5
ASIVAGISGQ +
b7
y6 y5
ASIVAGISGQ ++
b7

e

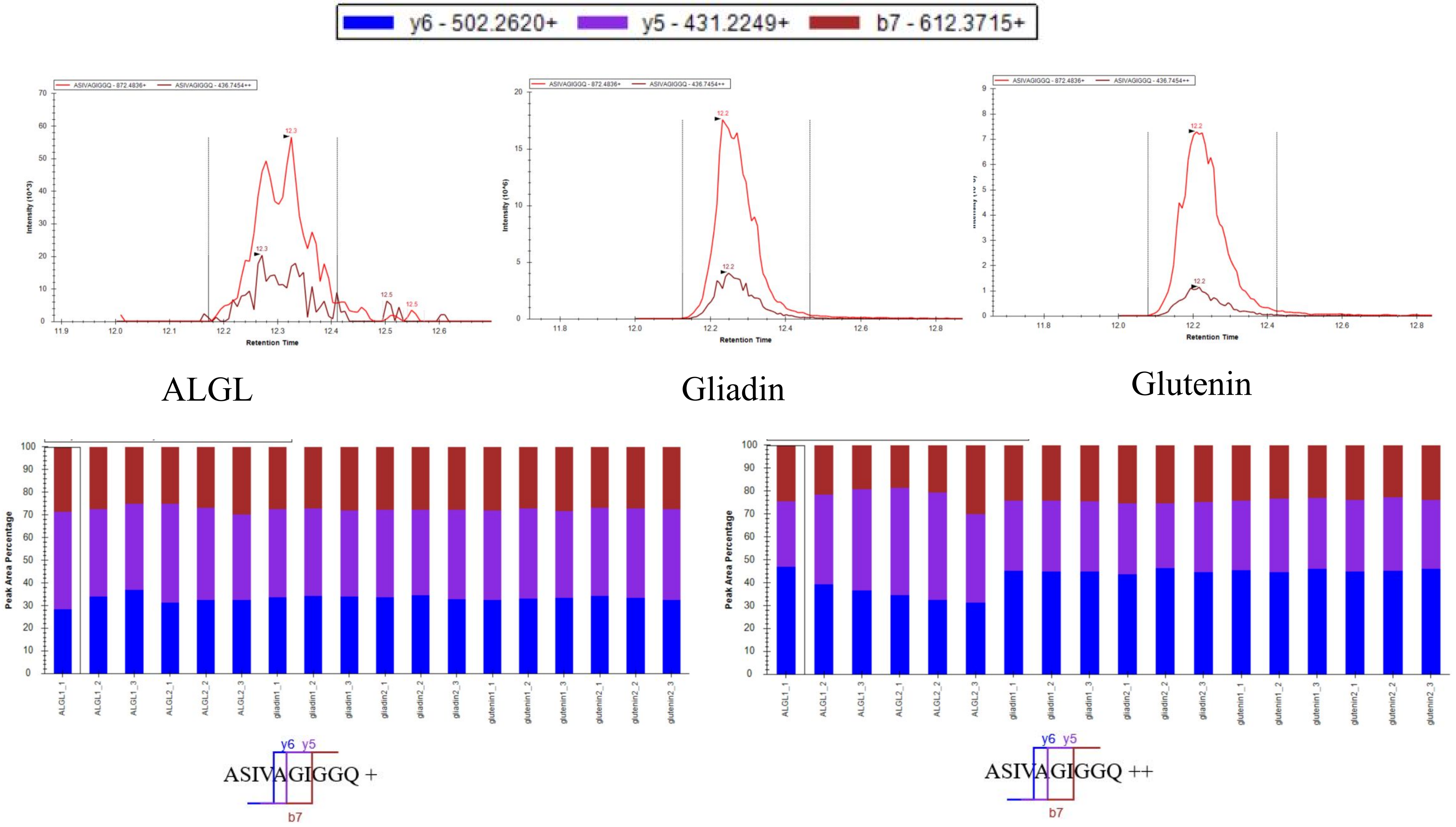
y6 - 502.2620+
y5 - 431.2249+
b7 - 612.3715+
ASIVAGIGGQ - 872.4836+
ASIVAGIGGQ - 436.7454++
Intensity (10^3)
Intensity (10^6)
Retention Time
ALGL
Gliadin
Glutenin
Peak Area Percentage
ALGL1_1
ALGL1_2
ALGL1_3
ALGL2_1
ALGL2_2
ALGL2_3
gliadin1_1
gliadin1_2
gliadin1_3
gliadin2_1
gliadin2_2
gliadin2_3
glutenin1_1
glutenin1_2
glutenin1_3
glutenin2_1
glutenin2_2
glutenin2_3
y6 y5
ASIVAGIGGQ +
b7
y6 y5
ASIVAGIGGQ ++
b7

f

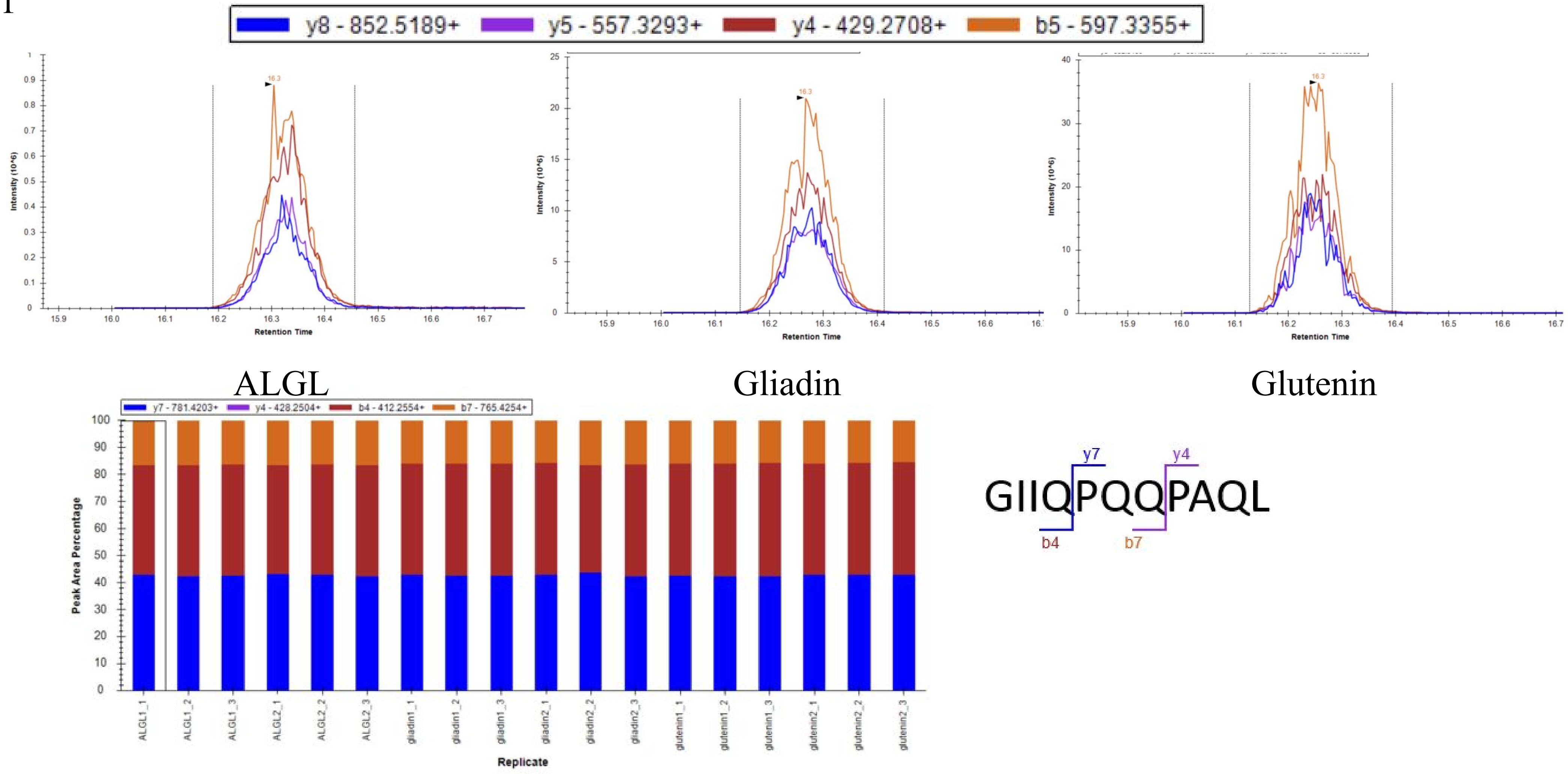

y8 - 852.5189+
y5 - 557.3293+
y4 - 429.2708+
b5 - 597.3355+
Intensity (10^6)
Retention Time
16.3
ALGL
Gliadin
Glutenin
y7 - 781.4203+
y4 - 428.2504+
b4 - 412.2554+
b7 - 765.4254+
Peak Area Percentage
Replicate
ALGL1_1
ALGL1_2
ALGL1_3
ALGL2_1
ALGL2_2
ALGL2_3
gliadin1_1
gliadin1_2
gliadin1_3
gliadin2_1
gliadin2_2
gliadin2_3
glutenin1_1
glutenin1_2
glutenin1_3
glutenin2_1
glutenin2_2
glutenin2_3
y7
y4
GIIQPQQPAQL
b4
b7

g

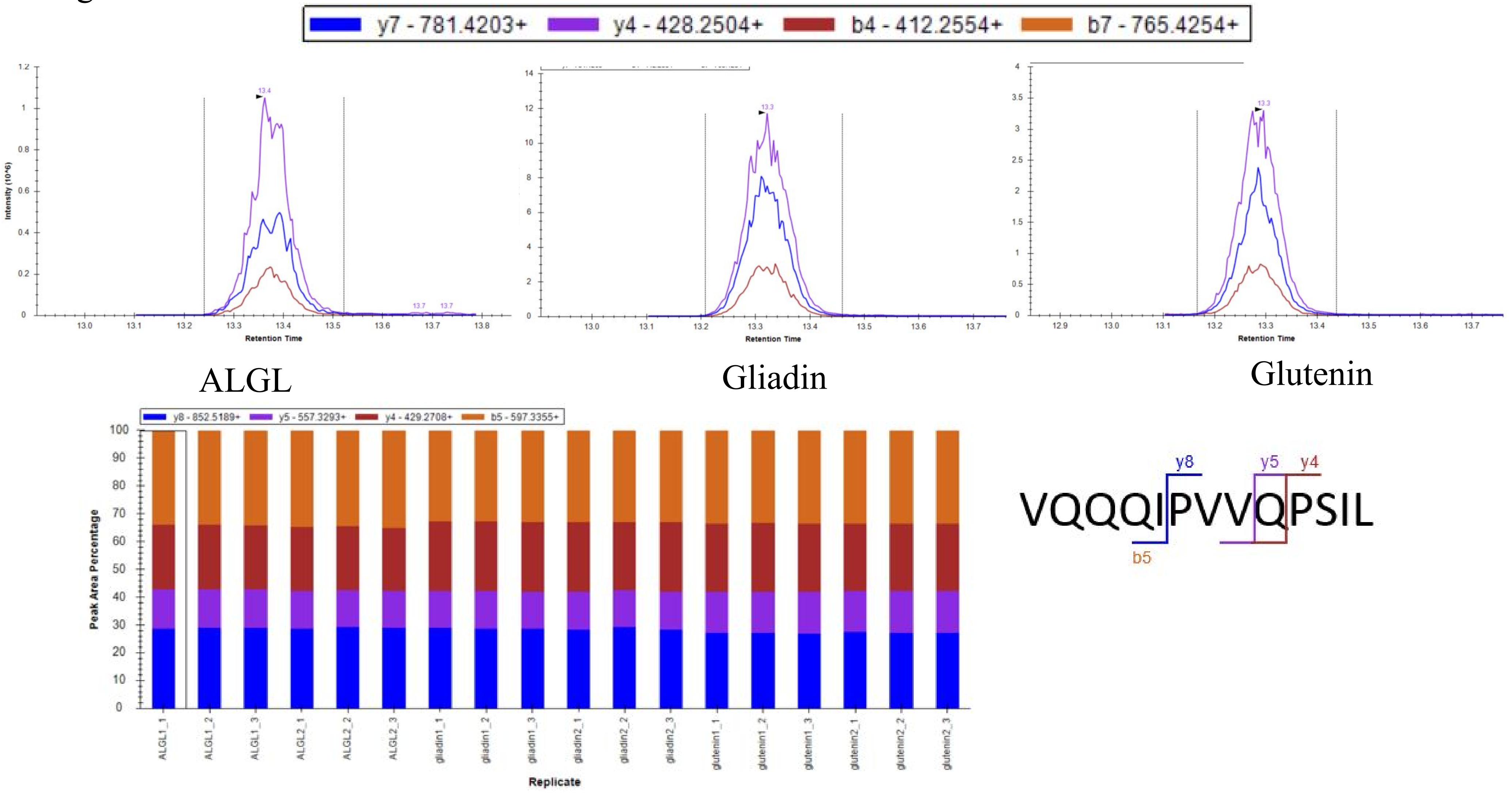

y7 - 781.4203+
y4 - 428.2504+
b4 - 412.2554+
b7 - 765.4254+
Intensity (10^6)
Retention Time
ALGL
Gliadin
Glutenin
y8 - 852.5189+
y5 - 557.3293+
y4 - 429.2708+
b5 - 597.3355+
Peak Area Percentage
Replicate
ALGL1_1
ALGL1_2
ALGL1_3
ALGL2_1
ALGL2_2
ALGL2_3
gliadin1_1
gliadin1_2
gliadin1_3
gliadin2_1
gliadin2_2
gliadin2_3
glutenin1_1
glutenin1_2
glutenin1_3
glutenin2_1
glutenin2_2
glutenin2_3
y8
y5
y4
VQQQIPVVQPSIL
b5

h

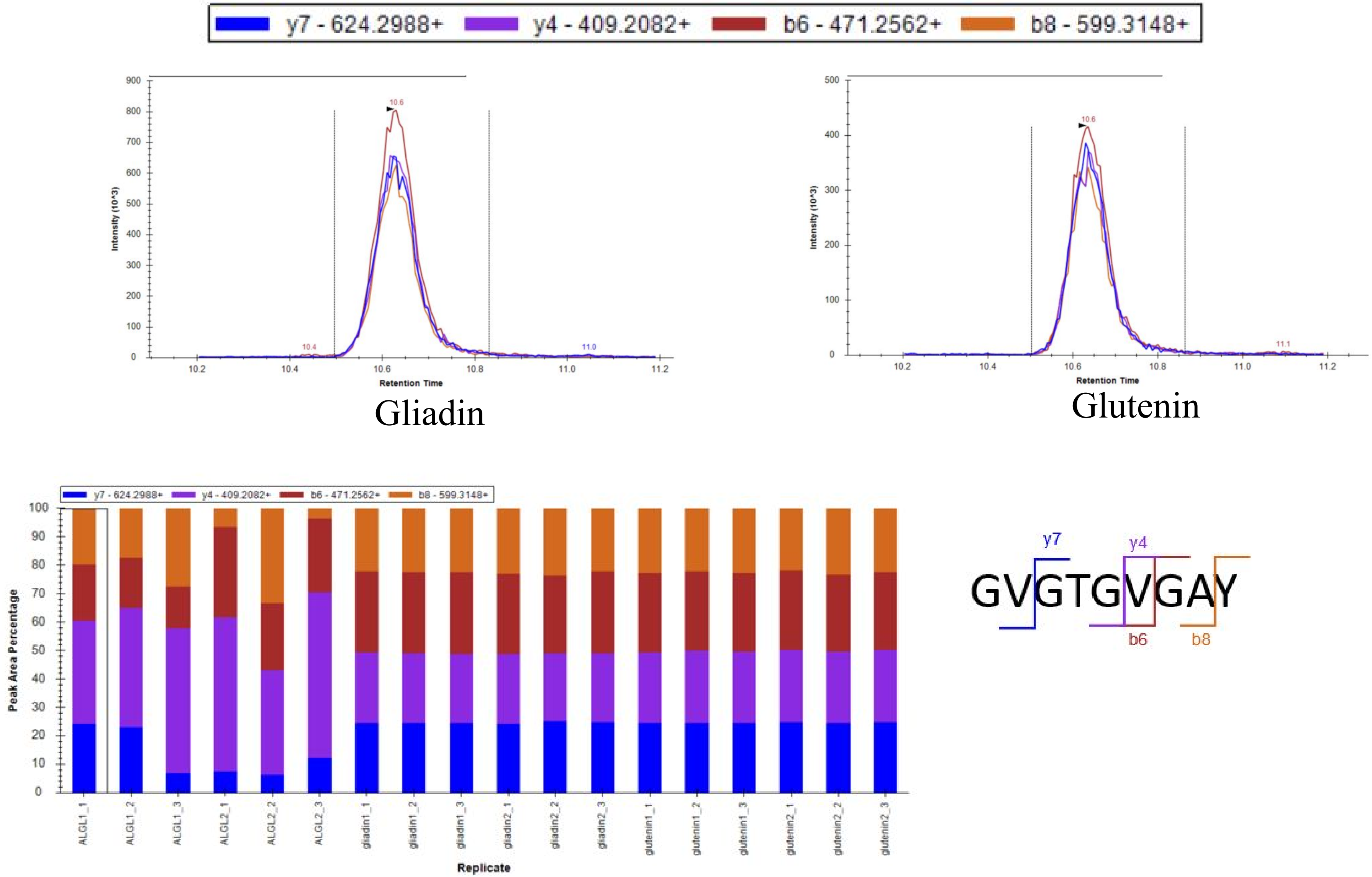

y7 - 624.2988+
y4 - 409.2082+
b6 - 471.2562+
b8 - 599.3148+
Intensity (10^3)
Retention Time
Gliadin
Glutenin
Peak Area Percentage
Replicate
ALGL1_1
ALGL1_2
ALGL1_3
ALGL2_1
ALGL2_2
ALGL2_3
gliadin1_1
gliadin1_2
gliadin1_3
gliadin2_1
gliadin2_2
gliadin2_3
glutenin1_1
glutenin1_2
glutenin1_3
glutenin2_1
glutenin2_2
glutenin2_3
GVGTGVGAY
y7
y4
b6
b8

i

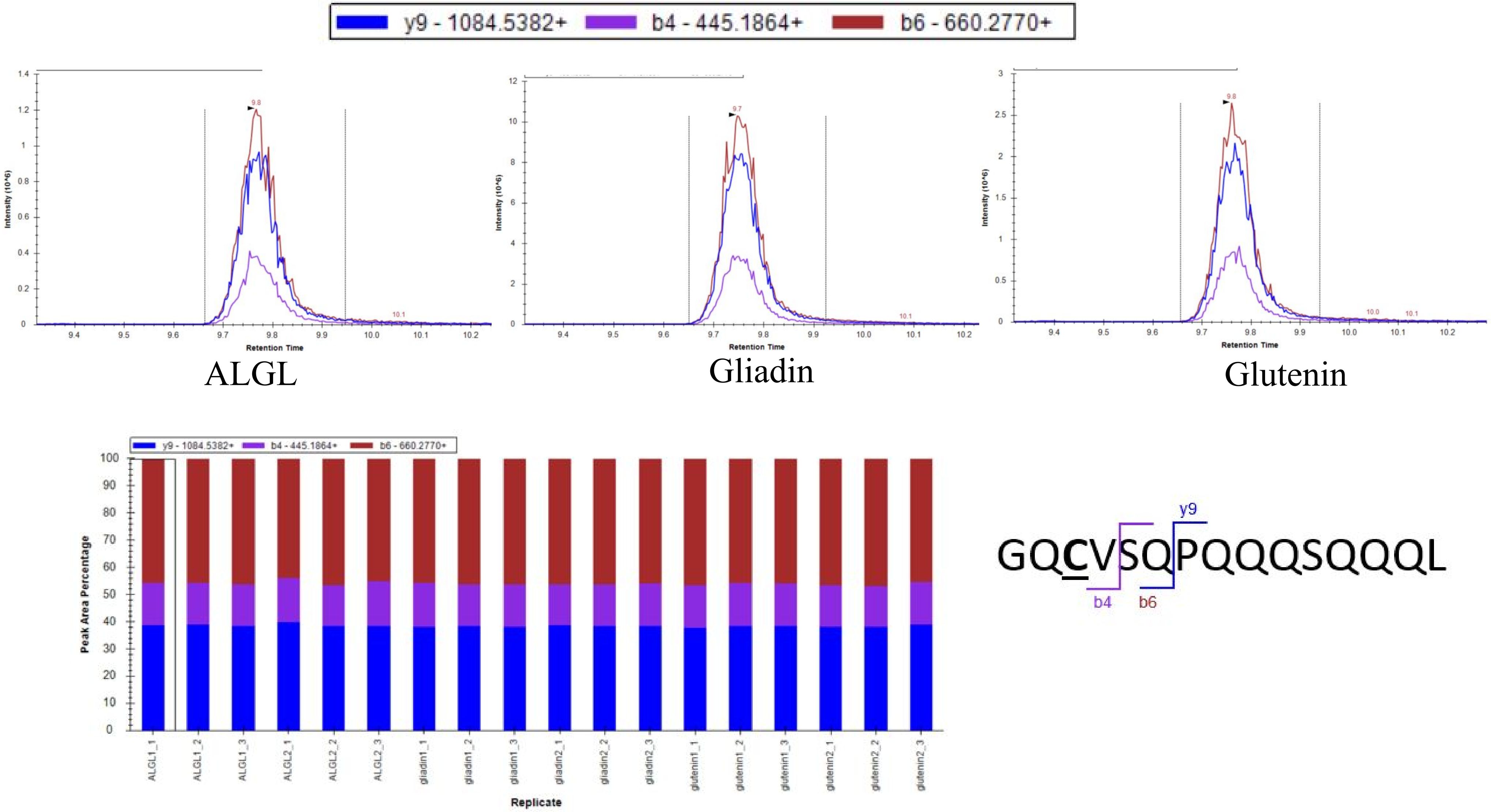

y9 - 1084.5382+
b4 - 445.1864+
b6 - 660.2770+
Intensity (10^6)
Retention Time
ALGL
Gliadin
Glutenin
Peak Area Percentage
Replicate
ALGL1_1
ALGL1_2
ALGL1_3
ALGL2_1
ALGL2_2
ALGL2_3
gliadin1_1
gliadin1_2
gliadin1_3
gliadin2_1
gliadin2_2
gliadin2_3
glutenin1_1
glutenin1_2
glutenin1_3
glutenin2_1
glutenin2_2
glutenin2_3
GQCVSQPQQSQQQL
y9
b4
b6

Figure. S3. The LC-MS chromatogram obtained from three fractions of peptide1-9 (a-i).

Peptide 1 showed good ionisation, as indicated by the sharp peak found in all fractions. Peptide 2, only ALGL can be detected. Peptide 3 showed good ionisation level similar as peptide 1. For peptide 4, similar double-peak patterns were observed in gliadin and glutenin fraction, especially in doubly charged state, while multiple unspecific peaks were identified in ALGL fraction. Although peptide 4 and 5 differed by only one amino acid residue, the ion transitions were very different, the singly charged peptides occupied nearly one-fold of intensity of the doubly charged peptide 5. Both gliadin and glutenin fraction shared double peaks against peptide 8, however, the presence of the second peak, which might be cause by the formation of isoforms. Peptide 9 showed nice and steady transition among the fractions.

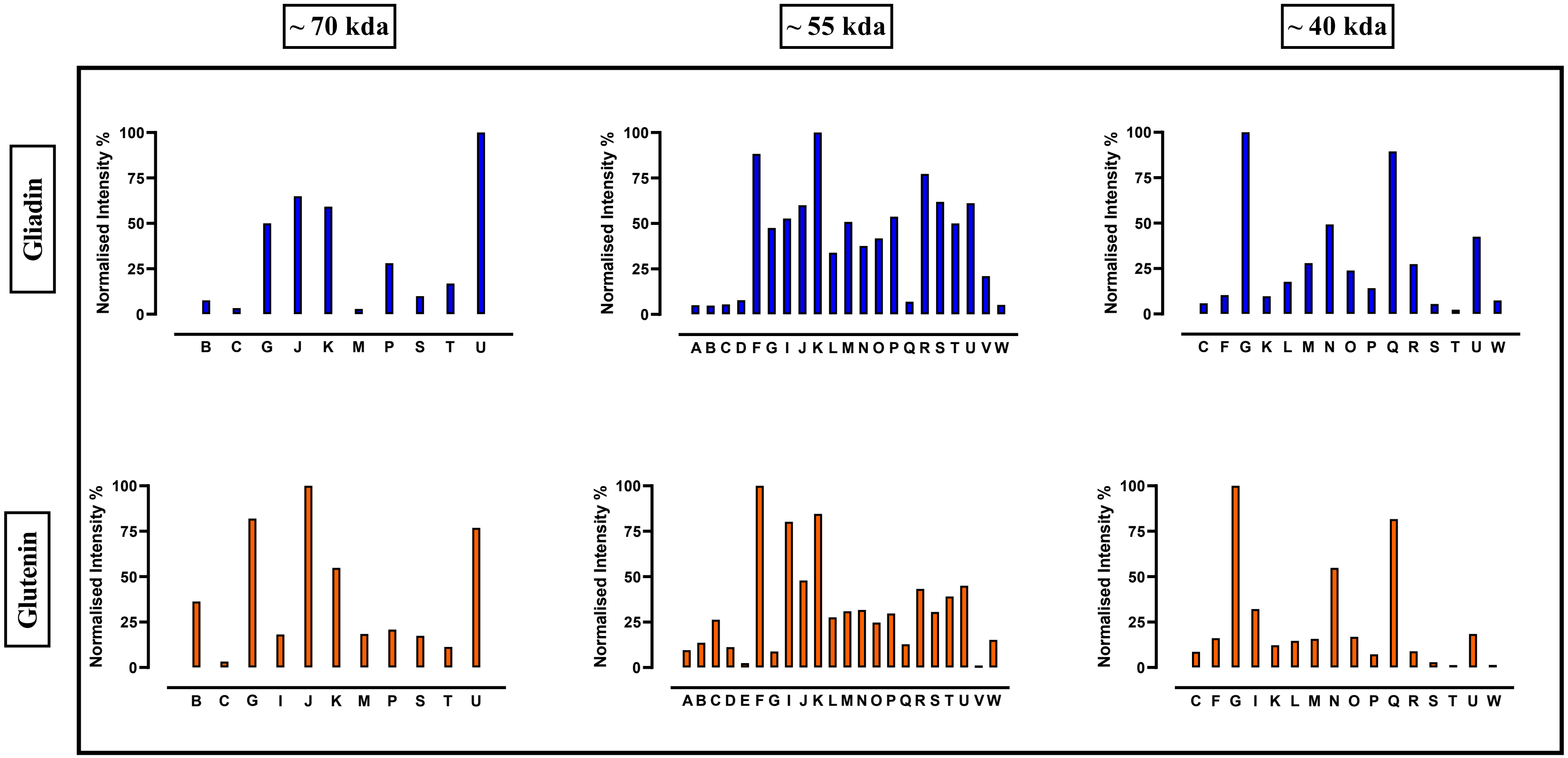

Figure. S4. The normalised binding intensity identified from IgE immunoblots of gliadin and glutenin at Mr ~ 70, 55, and 40 kDa

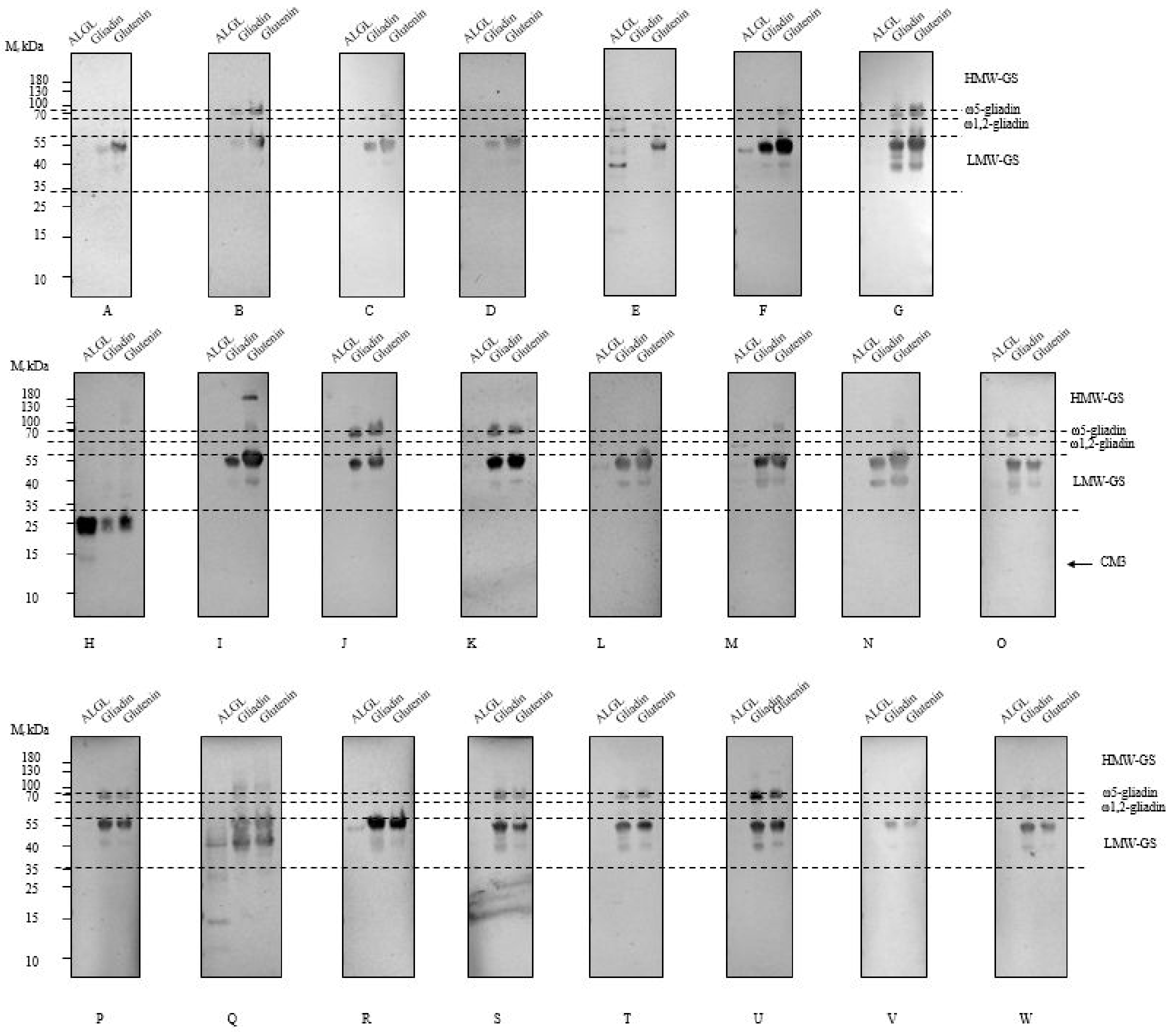

Figure. S5. The IgE-reactivity of the Osborne fractions against patients with IgE-mediated wheat allergy (sera A-W).

a

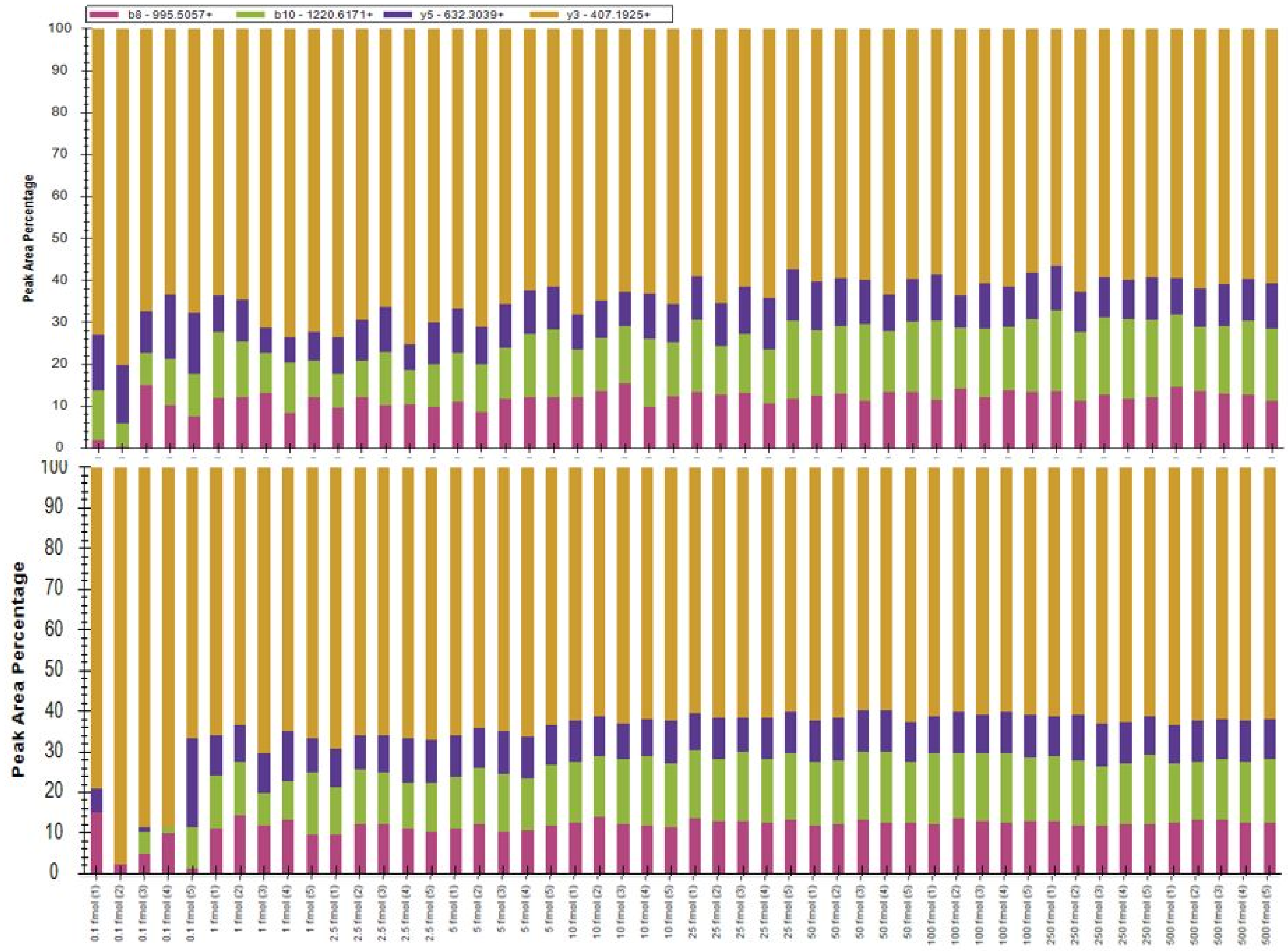
b8 - 995.5057+
b10 - 1220.6171+
y5 - 632.3039+
y3 - 407.1925+
Peak Area Percentage
Peak Area Percentage

b

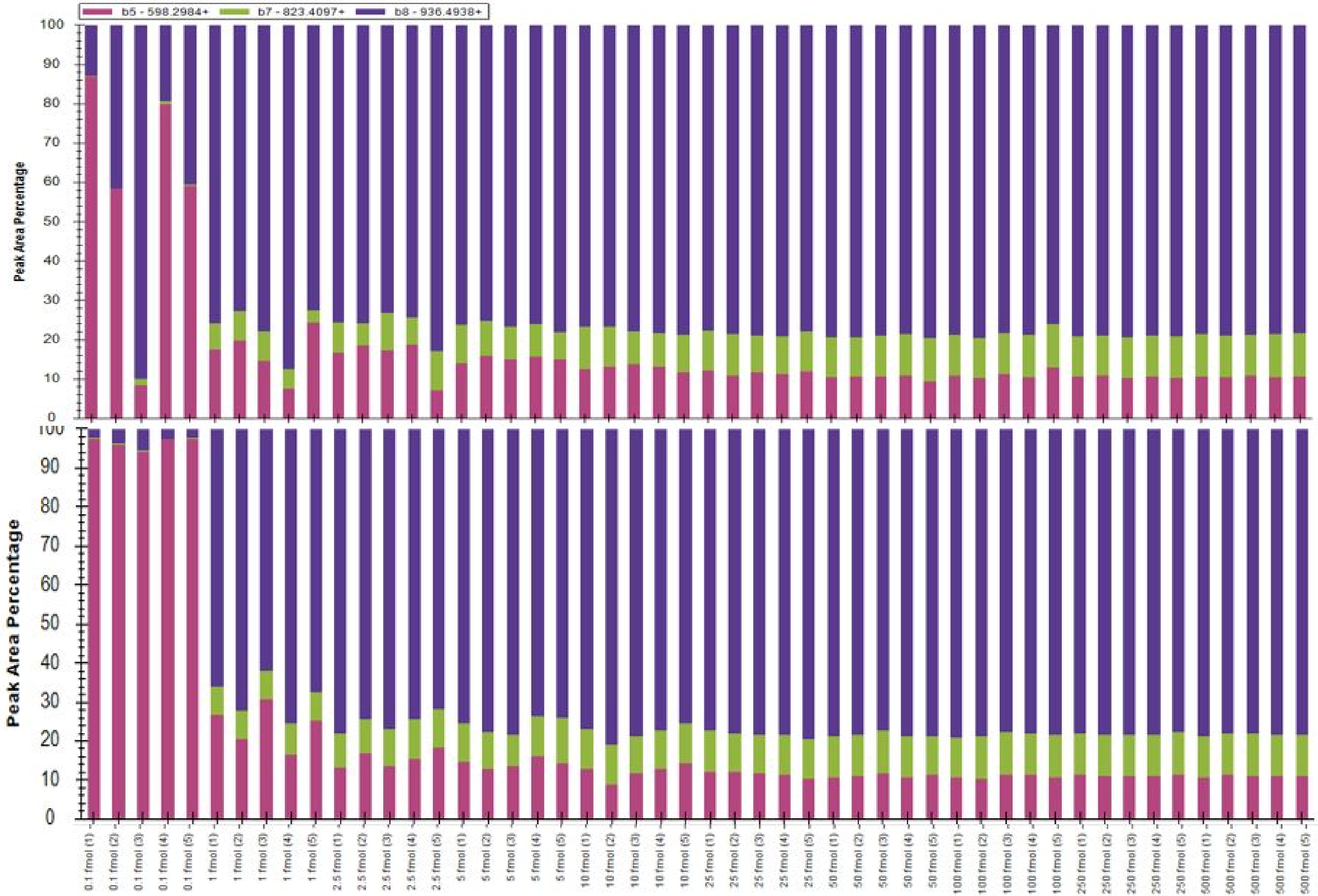

b5 - 598.2984+
b7 - 823.4097+
b8 - 936.4938+
Peak Area Percentage
Peak Area Percentage

c

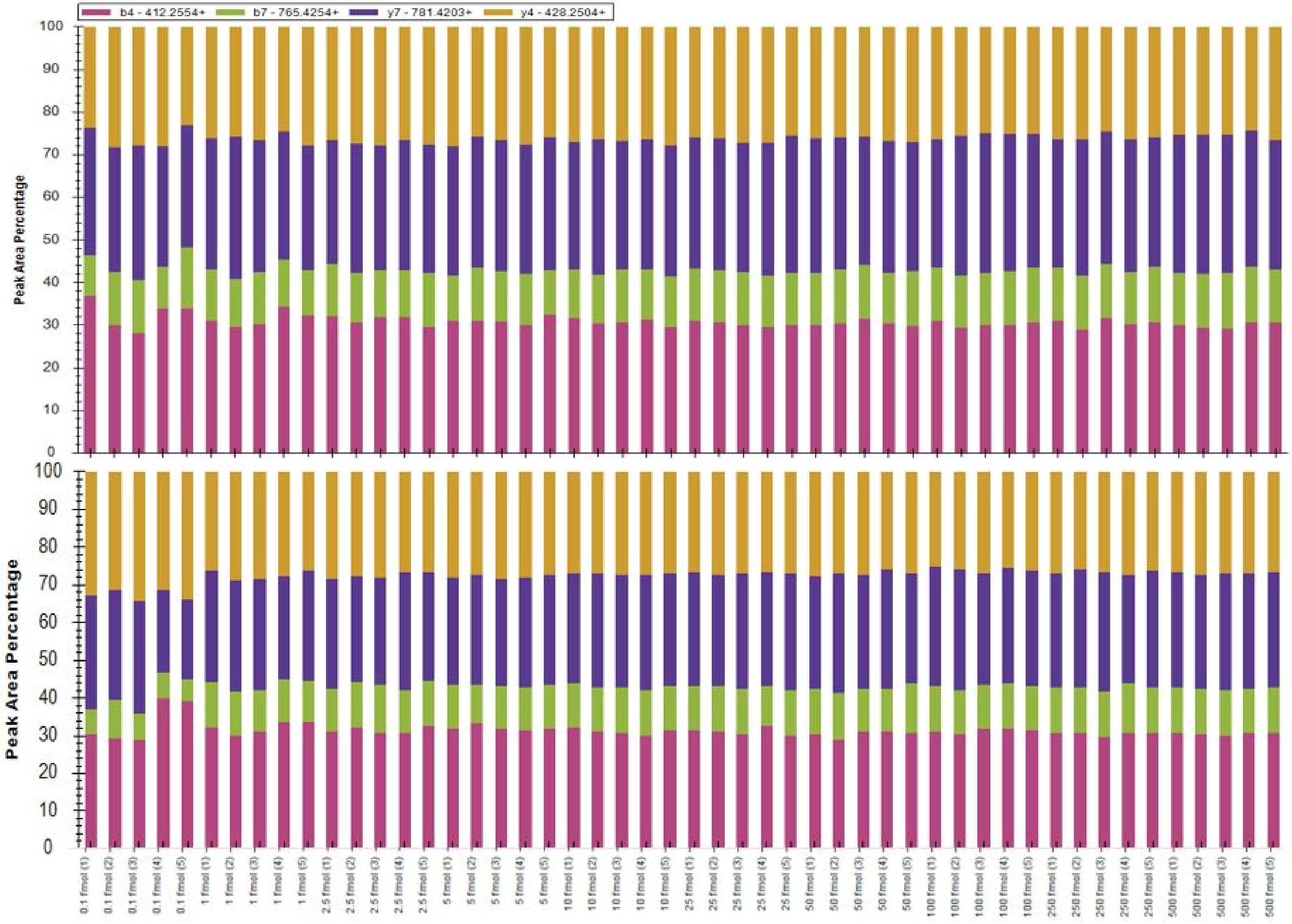
b4 - 412.2554+
b7 - 765.4254+
y7 - 781.4203+
y4 - 428.2504+
Peak Area Percentage
Peak Area Percentage

d

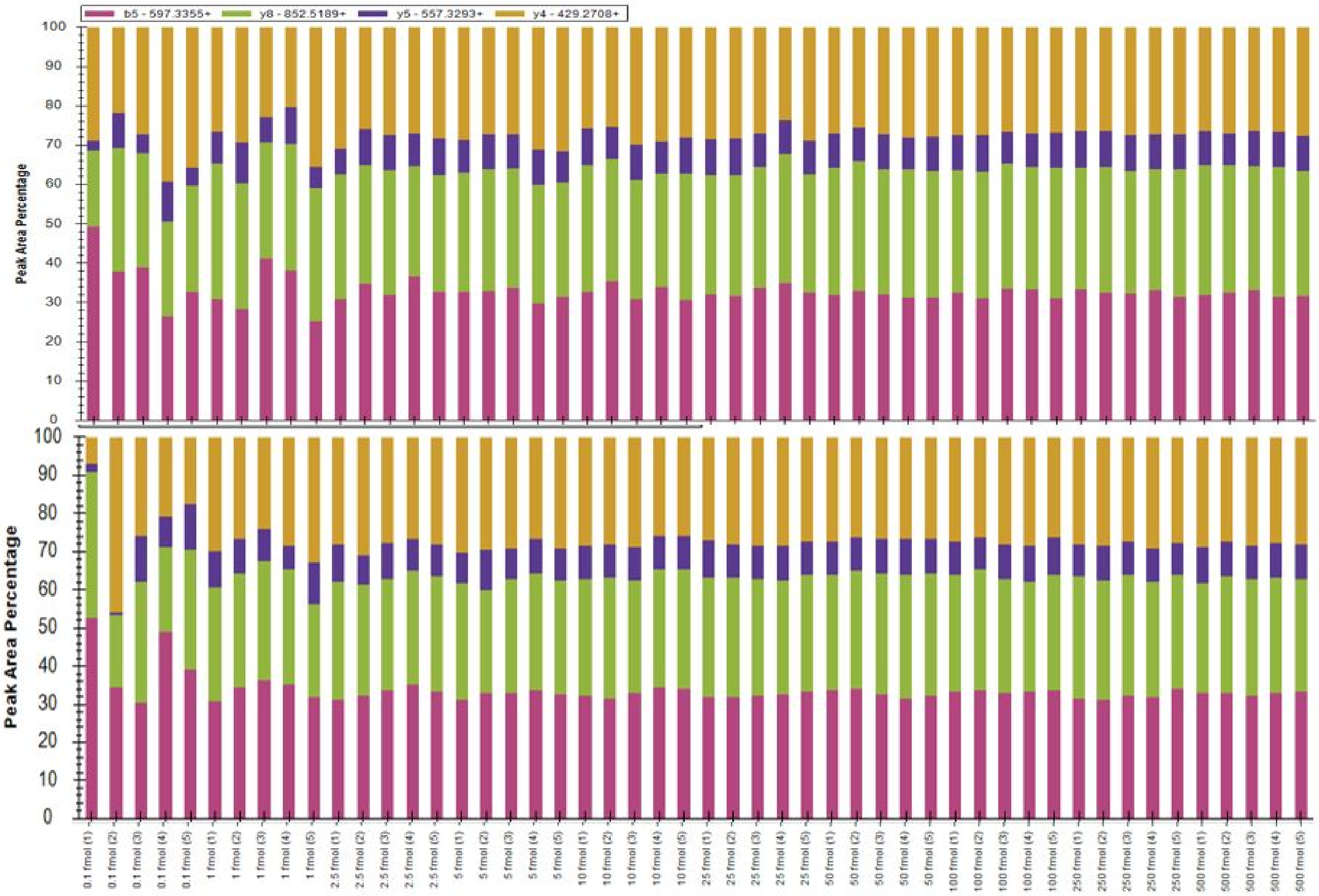

Figure. S6. Ion transition peptides in buffer (top) and in gluten-free flour matrix (bottom). RPQQPYPQPQPQY (a), QPFPQPQLPY (b), GIIQPQQPAQL (c), and VQQQIPVVQPSIL (d). Peptide dilutions ranged from 0.1 to 500 fmol on column.

Supplementary results S1 HPLC characterisation of protein fractions

The Osborne fractions were further characterised by RP-HPLC, and peak fractions were subjected to further analysis by SDS-PAGE (Fig. 3-5). The ALGL fraction showed a complex pattern of peaks eluting from 4 to 16 min (Fig. 3a). SDS-PAGE of pooled fraction I showed no visible protein bands suggesting it contained other types of components that absorb at 210 nm wavelength (Fig. 3b). Fraction II was resolved into two peaks eluting between 8-12 min, which were shown by SDS-PAGE to contain polypeptides of M_r ~ 12 kDa polypeptide which probably corresponded to ATIs Two smaller peaks which eluted from 12 -16 min (fraction III) comprised several faint bands of M_r ~ 55 kDa and M_r ~ 14-20 kDa.

RP-HPLC analysis of the gliadin fraction (Fig. 4) showed a series of peaks characteristic of ω-, a- and γ-gliadins, as previously described [5]. Peak fractions (I-VII) were analysed by SDS-PAGE (Fig. 4b). Peak I showed the presence of M_r ~ 36-45 kDa proteins which eluted at around 4 min and are likely to correspond to a- and γ-gliadins. Peaks II and III had elution times consistent with their containing ω-gliadins [5, 6] but SDS-PAGE showed only faintly staining protein bands [7]. The remaining peak fractions (IV-VII) eluted between 10-16 min comprised α- and γ-gliadins, based on their elution times and molecular mass (M_r ~30-45 kDa) determined by SDS-PAGE. Analysis of the PWG-gliadin gave only poorly resolved peaks and consequently no peak fractions were collected for analysis by SDS-PAGE (Fig. S3)

The RP-HPLC chromatogram of glutenin (Fig. 5) showed an intense series of peaks eluting at 4-6 min. These were pooled and separated by SDS-PAGE (I) which showed bands of Mr ~80-120 kDa, consistent with their being HMW-GS (Fig. 5b). A poorly resolved series of peaks eluting at 8-12 min (pooled peaks II) were also shown by SDS-PAGE to contain HMW-GS. The LMW-GS and possibly some polymeric gliadin components were eluted from 12-16min with bands at Mr ~30-45 kDa observed on SDS-PAGE for pooled peaks III-VI (Fig. 5b).

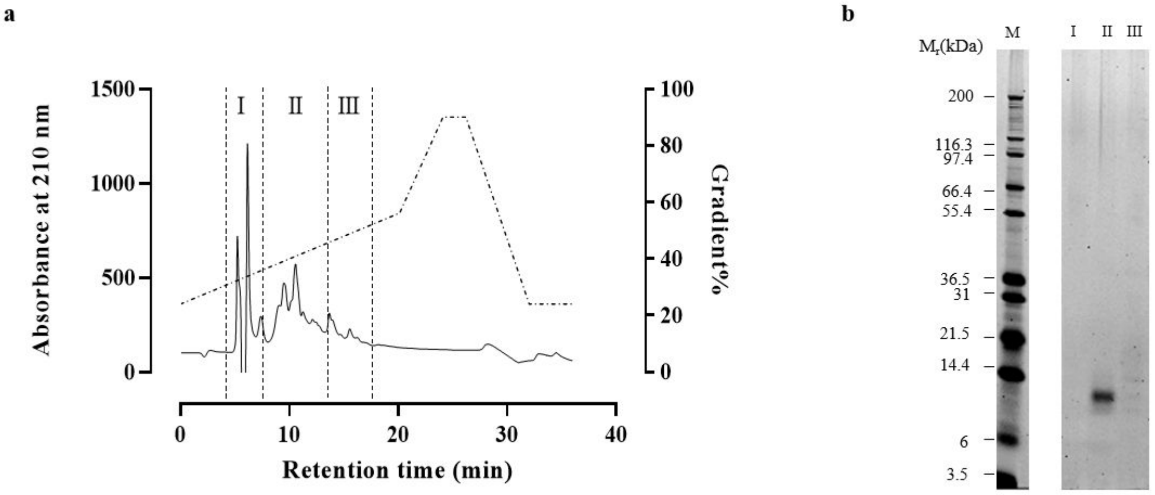

Figure. S7. RP-HPLC analysis of the ALGL fraction.

a) RP-HPLC chromatogram with the gradient shown as the black dashed line and showing the position of pooled peaks I-III b) SDS-PAGE of the pooled peaks: I - 0-8 min; II - 8-12 min; III: 12-16 min.

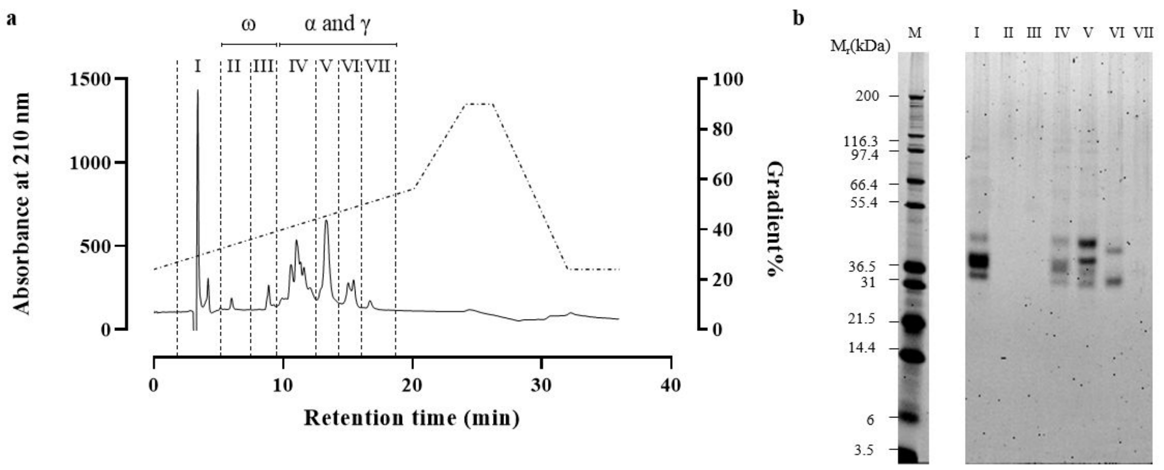

Figure. S8. RP-HPLC analysis of the gliadin fraction.

a) RP-HPLC chromatogram of gliadin fraction, the gradient shown as the black dashed line and showing the position of pooled peaks I-VII b) SDS-PAGE of the pooled peaks as follows: I – 0-5 min; II – 5-7 min; III – 7-9 min; IV – 9-12.5 min; V – 12.5-14 min; VI – 14-16 min; VII – 16-18 min.

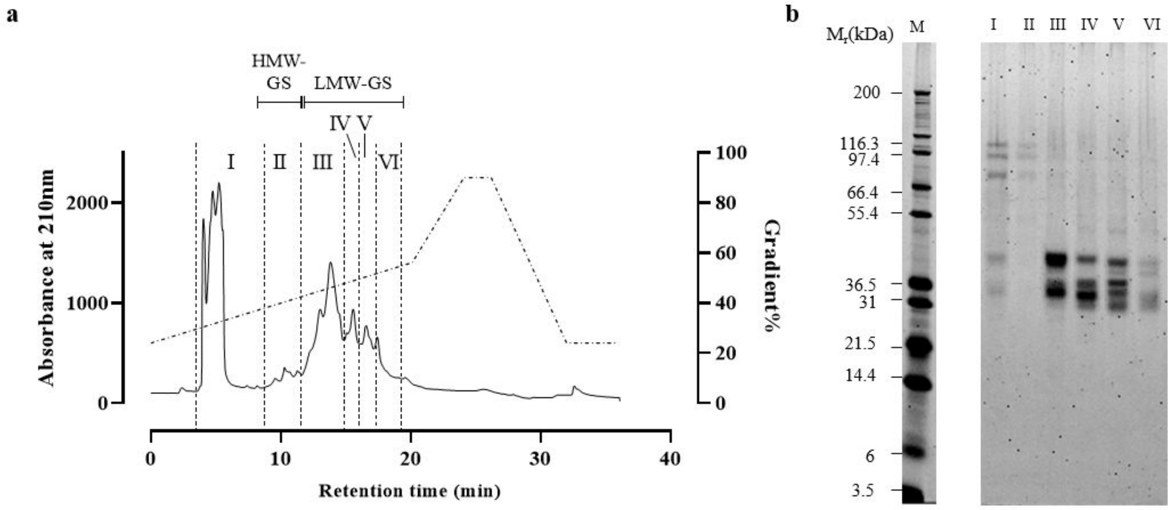

Figure. S9. RP-HPLC analysis of the glutenin fraction.

a) RP-HPLC chromatogram of the glutenin fraction, the gradient shown as the black dashed line and showing the position of pooled peaks I-VI b) SDS-PAGE of pooled peaks as follows: I - 0-8 min; II - 8-12 min; III - 12-15 min; IV - 15-16.5 min; V - 16.5-17.5 min; VI - 17.5-20 min.

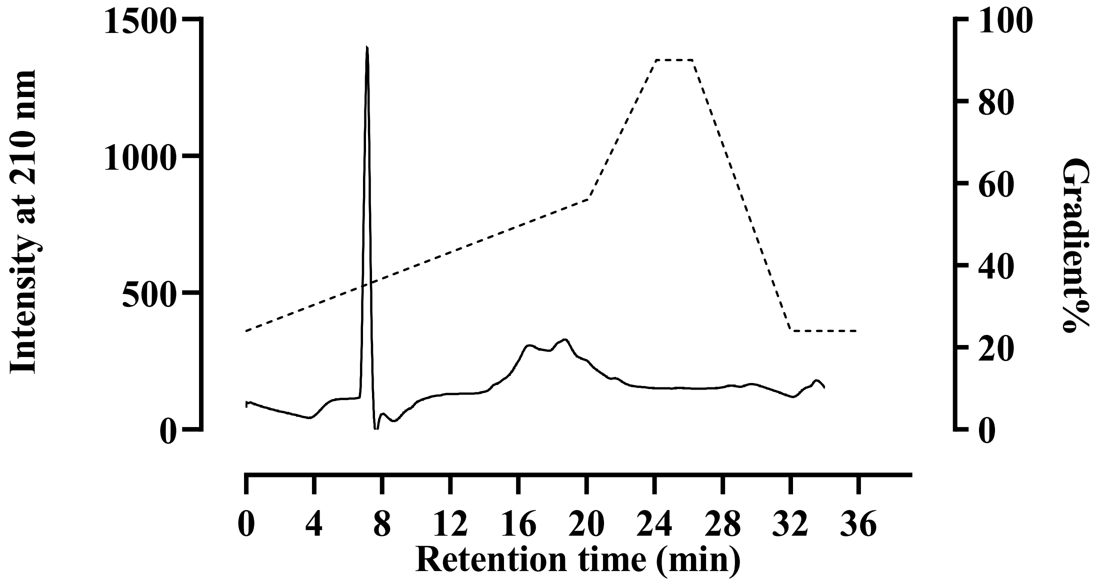

Figure. S10. The Chromatogram of PWG-gliadin.

The RP-HPLC chromatogram of PWG-gliadin, left y-axis showed the measured intensity at 210 nm, the black line showed the gradient of the acetonitrile with 0.1% FA on the right y-axis.

Supplementary References

1. Kahlenberg, F.; Sanchez, D.; Lachmann, I.; Tuckova, L.; Tlaskalova, H.; Méndez, E.; Mothes, T., Monoclonal antibody R5 for detection of putatively coeliac-toxic gliadin peptides. *European Food Research and Technology* **2006,** *222* (1), 78-82.
2. Morón, B.; Cebolla, A.; Manyani, H.; Alvarez-Maqueda, M.; Megías, M.; Thomas, M. d. C.; López, M. C.; Sousa, C., Sensitive detection of cereal fractions that are toxic to celiac disease patients by using monoclonal antibodies to a main immunogenic wheat peptide. *The American journal of clinical nutrition* **2008,** *87* (2), 405-414.
3. Brett, G.; Mills, E.; Goodfellow, B.; Fido, R.; Tatham, A.; Shewry, P.; Morgan, M., Epitope mapping studies of broad specificity monoclonal antibodies to cereal prolamins. *Journal of Cereal Science* **1999,** *29* (2), 117-128.
4. Mills, E. N.; Field, J. M.; Kauffman, J. A.; Tatham, A. S.; Shewry, P. R.; Morgan, M. R., Characterization of a monoclonal antibody specific for HMW subunits of glutenin and its use to investigate glutenin polymers. *Journal of agricultural and food chemistry* **2000,** *48* (3), 611-7.
5. Schalk, K.; Lexhaller, B.; Koehler, P.; Scherf, K. A., Isolation and characterization of gluten protein types from wheat, rye, barley and oats for use as reference materials. *PLOS ONE* **2017,** *12* (2), e0172819.
6. Wieser, H., Comparative investigations of gluten proteins from different wheat species I. Qualitative and quantitative composition of gluten protein types. *European Food Research and Technology* **2000,** *211* (4), 262-268.
7. van den Broeck, H. C.; America, A. H.; Smulders, M. J.; Gilissen, L. J.; van der Meer, I. M., Staining efficiency of specific proteins depends on the staining method: wheat gluten proteins. *Proteomics* **2008,** *8* (9), 1880-1884.
